# tRNA 2′-*O*-methylation by a duo of TRM7/FTSJ1 proteins modulates small RNA silencing in *Drosophila*

**DOI:** 10.1093/nar/gkaa002

**Published:** 2020-01-16

**Authors:** Margarita T Angelova, Dilyana G Dimitrova, Bruno Da Silva, Virginie Marchand, Caroline Jacquier, Cyrinne Achour, Mira Brazane, Catherine Goyenvalle, Valérie Bourguignon-Igel, Salman Shehzada, Souraya Khouider, Tina Lence, Vincent Guerineau, Jean-Yves Roignant, Christophe Antoniewski, Laure Teysset, Damien Bregeon, Yuri Motorin, Matthias R Schaefer, Clément Carré

**Affiliations:** 1 Transgenerational Epigenetics & small RNA Biology, Sorbonne Université, Centre National de la Recherche Scientifique, Laboratoire de Biologie du Développement - Institut de Biologie Paris Seine, 9 Quai Saint Bernard, 75005 Paris, France; 2 Next-Generation Sequencing Core Facility, UMS2008 IBSLor CNRS-Université de Lorraine-INSERM, BioPôle, 9 avenue de la Forêt de Haye, 54505 Vandoeuvre-les-Nancy, France; 3 Eucaryiotic Translation, Sorbonne Université, CNRS, Institut de Biologie Paris Seine, Biological Adaptation and Ageing, Institut de Biologie Paris Seine, 9 Quai Saint bernard, 75005 Paris, France; 4 Ingénierie Moléculaire et Physiopathologie Articulaire, UMR7365, CNRS - Université de Lorraine, 9 avenue de la Forêt de Haye, 54505 Vandoeuvre-les-Nancy, France; 5 Institut de Chimie de Substances Naturelles, Centre de Recherche de Gif CNRS, 1 avenue de la Terrasse, 91198 Gif-sur-Yvette, France; 6 Institute of Molecular Biology, Ackermannweg 4, 55128, Mainz, Germany; 7 Center for Integrative Genomics, Génopode Building, Faculty of Biology and Medicine, University of Lausanne, CH-1015, Lausanne, Switzerland; 8 ARTbio Bioinformatics Analysis Facility, Sorbonne Université, CNRS, Institut de Biologie Paris Seine, 9 Quai Saint Bernard, 75005 Paris, France; 9 Division of Cell and Developmental Biology, Center for Anatomy and Cell Biology, Medical University of Vienna, Schwarzspanierstrasse 17, A-1090 Vienna, Austria

## Abstract

2′-*O*-Methylation (Nm) represents one of the most common RNA modifications. Nm affects RNA structure and function with crucial roles in various RNA-mediated processes ranging from RNA silencing, translation, self *versus* non-self recognition to viral defense mechanisms. Here, we identify two Nm methyltransferases (Nm-MTases) in *Drosophila melanogaster* (CG7009 and CG5220) as functional orthologs of yeast TRM7 and human FTSJ1. Genetic knockout studies together with MALDI-TOF mass spectrometry and RiboMethSeq mapping revealed that CG7009 is responsible for methylating the wobble position in tRNA^Phe^, tRNA^Trp^ and tRNA^Leu^, while CG5220 methylates position C32 in the same tRNAs and also targets additional tRNAs. *CG7009* or *CG5220* mutant animals were viable and fertile but exhibited various phenotypes such as lifespan reduction, small RNA pathways dysfunction and increased sensitivity to RNA virus infections. Our results provide the first detailed characterization of two TRM7 family members in *Drosophila* and uncover a molecular link between enzymes catalyzing Nm at specific tRNAs and small RNA-induced gene silencing pathways.

## INTRODUCTION

The existence of RNA modifications has been known for over 50 years and many of the pioneering studies addressed the function of RNA modifications in abundantly expressed RNAs such as transfer RNAs (tRNAs) and ribosomal RNAs (rRNAs). tRNAs are the most heavily modified RNAs (up to 25% nucleotides/tRNA, (1)). tRNAs are modified post-transcriptionally and the biosynthesis of modified nucleosides requires different modification enzymes acting sometimes sequentially at distinct steps of tRNA maturation (2,3). The complex mechanisms underlying the stepwise modification of tRNAs were largely deciphered in the yeast *Saccharomyces cerevisiae*, as well as in studies conducted in prokaryotes and *Archaea*. More recently, some of the seminal findings in single-cell organisms were revisited using multi-cellular models, including studies that aim at understanding how mutations in tRNA modification enzymes affect organismal development and disease etiology.

2′-*O*-Methylation (Nm) is a common RNA modification. The addition of a methyl group to the 2′ hydroxyl of the ribose moiety of a nucleoside creates Nm (reviewed in (4,5)). Nm can occur at any nucleotide explaining the abundant nature of this modification. Nm residues are found at multiple and often highly conserved positions in tRNAs, rRNAs, and small nuclear RNAs (snRNAs) (6–8). In eukaryotes, RNA modification reactions resulting in Nm on rRNAs and snRNAs are frequently catalyzed by evolutionarily conserved C/D-box small RNAs (SNORDs) involving guide ribonucleoprotein particles (RNPs) which contain the Nm-methylase fibrillarin. Small nucleolar RNPs (snoRNPs) mediate the deposition of Nm at rRNAs (9–12) while small Cajal bodies RNPs (scaRNPs) direct Nm-modification to snRNAs (13–17). In contrast, most of the Nm deposition occurring in eukaryotic tRNAs is mediated by stand-alone proteins without the need for guidance by small RNAs. However, recently it was reported that one snoRNA and one scaRNA can guide Nm deposition to tRNA^Met^ in mammalian cells (18). Importantly, Nm deposition occurs also at 3′-terminal nucleotides in small non-coding RNAs (sncRNAs) such as microRNAs (miRNAs) and small-interfering RNAs (siRNAs) in plants (19–21), in Argonaute-2 (Ago2)-loaded siRNAs and miRNAs in flies and in PIWI-interacting RNAs (piRNAs) in animals (22–24). More recently, Nm was also reported to be internally deposited in messenger RNA (mRNA) (25–27).

Nm can affect RNAs in multiple ways as it increases hydrophobicity, protects RNAs from nuclease degradation (18,24,28), stabilizes helical structures or modulates interactions with proteins or other RNAs (29–40).

An important variety of tRNA modifications are deposited at the wobble position N_34_ in the anticodon loop (ACL), and at the anticodon-adjacent position N_37_. Among the different tRNA isoacceptors, these two positions contain highly conserved modifications, which is suggestive of their physiological importance. Accordingly, it was shown that ACL modifications prevented frameshifting during translation (41,42) and are thus necessary for the correct decoding of genetic information (43).

The methyltransferase complex catalysing Nm formation in the ACL of mammalian and yeast tRNAs comprises the Nm-methyltransferases (Nm-MTases) FTSJ1 or TRM7, respectively. These enzymes associate with specific proteins: THADA/TRM732 for Nm_32_ and WDR6/TRM734 for Nm_34_ formation (2,44,45). Cm_32_ and, more importantly Nm_34_, are required for efficient formation of a third modification, wybutosine (yW) at m^1^G_37_ in tRNA^Phe^ (46–48). The same sequential circuitry is conserved in the phylogenetically distant yeast *Schizosaccharomyces pombe* (45), while the formation of peroxywybutosine (o2yW) at position 37 is also affected in humans lacking FTSJ1 (45,49).

Several studies have uncovered crucial roles for FTSJ1/TRM7 in normal and pathological conditions (reviewed in (4,5,50)). While in *S. cerevisiae* and *S. pombe*, lack of TRM7 affected translational regulation and growth (48,51,52), *FTSJ1* mutant mice showed impairment in their learning capacity, as well as significantly reduced pain sensing (hypoalgesia) and altered gene expression profiles (53). Similarly, in humans, several mutations in *FTSJ1* were shown to be causative of a neurodevelopmental disorder known as Non-Syndromic X-linked Intellectual Disability (NSXLID) (49,53–55). Importantly, expression of human FTSJ1 in yeast suppressed the severe growth defects observed in *trm7Δ* mutants, demonstrating that the TRM7 enzyme family and their RNA targets are highly conserved (45).

While the molecular function of yeast and human Nm-MTases acting on specific tRNAs has been established, the molecular mechanisms causing the complexity of observed mutant phenotypes have not been fully elucidated. Importantly, a tractable multicellular model system that would allow studying Nm-MTase function systematically and thereby bridge the growth phenotypes observed in *trm7* deficient yeast with the complex phenotypes observed in *FTSJ1*-mutant human has been lacking.

In this report, we show that, in contrast to yeast and humans, *Drosophila melanogaster* has evolved two Nm-MTase genes, *CG5220* and *CG7009*, whose products specialized their activity to respectively methylate positions 32 and 34 in the ACL of specific tRNAs. We demonstrate that the catalytic specificity of these Nm-MTases is dependent on the position rather than the identity of the ACL nucleotides. Importantly, lack of these proteins reduced *Drosophila* lifespan and impaired various cellular pathways, which employ small RNAs to achieve post-transcriptional silencing. Hence, *CG5220* and *CG7009* mutant animals were more sensitive to RNA virus infections and showed dysfunctional control of transposable elements, suggesting a molecular link between Nm RNA modifications and small RNA gene silencing pathways in *Drosophila*.

## MATERIALS AND METHODS

### Automig DRSC 2.0 genome-wide screening library

We screened 22.490 dsRNA of 400 bp length on average allowing the inactivation of 94.3% of all annotated *Drosophila* genes, including predicted genes (library version DRSC 2.0). About 13 900 genes are represented by the collection (∼66 assay plates), targeted on average by one to two dsRNAs per gene. More information about the DRSC Genome-wide RNAi Library (DRSC 2.0) can be found at the DRSC/TRiP Functional Genomics Resources. The recommended protocol was followed (56) on seven series of duplicated 384-well plates, which were screened over a period of three weeks. Briefly, 250 ng/well (5 μl at 50 ng/μl) of each dsRNAs were distributed into 384-well culture plates in 62 plates. One plate is organized in 16 rows (A-P) and 24 columns (1–24). Each well thus possesses a unique identification number consisting of the plate number followed by well coordinates. Each dsRNA has a unique identification number (DRSC#####). A *WellMate* dispenser (Matrix Technologies) was used to distribute the *automiG* S2R+ cell culture suspension into the 384-well plates (25.000 cells at a concentration of 2.5 × 10^6^ cells/ml). After dsRNA internalization into the cells for one hour, 30 μl of 10% heat-inactivated fetal calf serum was added per well. The cells were incubated for 4 days with the dsRNA before the expression of *automiG* in order to allow the complete internalization of the dsRNAs, the degradation of the target mRNAs and the catabolism of the corresponding protein. At day 5, *automiG* expression was induced with 600 μM of CuSO_4_/well. After 24 h of *automiG* induction, the cells were imaged on an *Opera* confocal microscope (Evotec Technologies, Perkin Elmer) using an automated acquisition system allowing fast imaging of the epifluorescence in each well of a large number of plates. In addition, Analyst^®^GT multimode reader (Molecular Devices) – a plate reader allowing the fast and sensible read-out of 40 plates per group was used.

A validation screen was performed in triplicate using the same conditions as those used in the primary screen described above. After a 48 hours incubation period, plates were centrifuged for one minute at 800 g and the culture medium was carefully removed. 25 μl of cracking buffer (125 mM Tris pH 6.8, 5% ß-mercapto-ethanol, 2% SDS, 4 M urea) was added in each well and 8 μl of protein extracts were analyzed by western blotting. Further information is available upon request and at the *DRSC/TRiP Functional Genomics Resources*, Harvard University.

### Amino Acid conservation and phylogenetic analysis

Sequence alignments and visualization were performed in Kalign (www.ebi.ac.uk/Tools/msa/kalign/) and Unipro UGENE 1.32.0. Percentage of amino acid (aa) identities and coverages between CG7009, CG5220, TRM7 and FTSJ1 proteins were determined on the Ensembl project website (www.ensembl.org). For phylogenetic analysis, protein alignments were performed using mafft v7.407 with default parameters (57). Removal of positions with >50% of gaps was obtained by using trimal v1.4 (58). Phylogenetic analysis was performed using raxml v8.2.12 (59) under the PROTGAMMALG model by combining a rapid bootstrap analysis (100 replicates) and search for the best ML tree (-f a option).

### Total RNA extraction for MALDI-TOF and RiboMethSeq analysis

3–5 days old females and males were homogenized on a Precellys 24^®^ tissue homogenizer (Bertin Technology) in 1 ml TRI-reagent/50 flies (Sigma Aldrich). Total RNA from 20 ml of fly lysates (1000 flies), was extracted with 8 ml of chloroform and precipitated with two-thirds volumes of isopropanol. The pellets were air-dried and resuspended in RNase-free water.

### Purification of tRNA^Phe(GAA)^

Total RNA preparations (∼7 mg) were supplemented with LiCl to a final concentration of 0.8 M and incubated overnight at 4°C to precipitate high-molecular mass molecules. The precipitate was eliminated by centrifugation and the supernatant was supplemented with two volumes of 100% ethanol and incubated at –20°C for two hours to precipitate small RNAs. After centrifugation, pelleted small RNAs were washed twice in 70% ethanol and resuspended in one ml of RNase-free water. tRNAs were further purified using the NucleoBond RNA/DNA 400 kit (Macherey-Nagel) following manufacturer's instructions, except that the elution step was performed with 5 ml of 100 mM Tris–acetate (pH 6.3); 15% ethanol and 600 mM KCl. Eluted tRNA were ethanol precipitated and resuspended in one ml of RNase-free water. Purification of tRNA^Phe(GAA)^ was performed using a 5′ biotinylated complementary oligonucleotide (5′-biotin-TGGTGCCGAAACCCGGGATTGAACCGGGG-3′) coupled to Streptavidin Magnesphere Paramagnetic particles (Promega). Annealing of specific tRNA was performed in 1× TMA buffer (Tris–HCl pH 7.5 10 mM, ethylenediaminetetraacetic acid (EDTA) 0.1 mM, tetramethylammonium chloride 0.9 M) after heating the mixture at 95°C for 3 min followed by cooling to 60°C for 30 min. Paramagnetic particles were washed three times with 1× TMA buffer and specific tRNA^Phe(GAA)^ was recovered by heating the final suspension at 95°C for 3 min. tRNA^Phe(GAA)^ was desalted and concentrated four times to 50 μl using Vivacon 500 devices (Sartorius; 2000 MWCO) using 100 mM ammonium acetate (pH 5.3) as a final buffer. The average yield obtained from 7 mg of total RNA was ∼2–7 μg of purified tRNA^Phe(GAA)^*. Note*: If used for RiboMethSeq, LiCl was washed away because of its interference with adaptor ligation during the cDNA library preparation.

### MALDI-TOF analysis of digested tRNA^Phe(GAA)^

For mass spectrometry analysis, ∼500 ng of tRNA^Phe(GAA)^ were digested with 100 units of RNase T1 (Sigma) in a final volume of 10 μl at 37°C for 4 h. RNase T1 cleaves the phosphodiester bond between the 3′-guanylic residue and the 5′-OH residue of adjacent nucleotides and generates 3′-phosphate nucleosides. One microliter of digest was mixed with 9 μl HPA (40 mg/ml in water:acetonitrile 50:50) and 1 μl of the mixture was spotted on the MALDI plate and air-dried (‘dried droplet’ method). MALDI-TOF MS analyses were performed directly on the digestion products using an UltrafleXtreme spectrometer (Bruker Daltonique, France). Acquisitions were performed in positive ion mode.

### RiboMethSeq

RiboMethSeq analysis of *D. melanogaster* tRNAs was performed as described in (60). Briefly, tRNAs extracted from whole flies were fragmented in 50 mM bicarbonate buffer pH 9.2 for 15 min at 95°C. The reaction was stopped by ethanol precipitation. The pellet was washed with 80% ethanol and sizes of generated RNA fragments were assessed by capillary electrophoresis using a small RNA chip on Bioanalyzer 2100 (Agilent, USA). RNA fragments were directly 3′-end dephosphorylated using 5 U of Antarctic Phosphatase (New England Biolabs, UK) for 30 min at 37°C. After inactivation of the phosphatase for 5 min at 70°C, RNA fragments were phosphorylated at the 5′-end using T4 PNK and 1 mM ATP for one hour at 37°C. End-repaired RNA fragments were then purified using RNeasy MinElute Cleanup kit (QIAGEN, Germany) according to the manufacturer's recommendations. RNA fragments were converted to library using NEBNext^®^ Small RNA Library kit (ref#E7330S, New England Biolabs, UK or equivalent from Illumina, USA) following the manufacturer's instructions. DNA library quality was assessed using a High Sensitivity DNA chip on a Bioanalyzer 2100. Library sequencing was performed on Illumina HiSeq 1000 in single-read mode for 50 nt. Primary analysis of sequencing quality was performed with RTA 2.12 software, to insure > Q30 quality score for >95% of obtained sequences.

Following SR50 sequencing run, demultiplexing was performed with BclToFastq v2.4, reads not passing quality filter were removed. Raw reads after demultiplexing were trimmed with Trimmomatic v0.32 (61). Alignment to the reference tDNA sequences was performed with bowtie 2 ver2.2.4 (62) in End-to-End mode. Uniquely mapped reads were extracted from *sam file by RNA ID and converted to *.bed format using bedtools v2.25.0 (63). Positional counting of 5′-and 3′-ends of each read was performed with awk Unix command. Further treatment steps were performed in R environment (v3.0.1). In brief, 5′-and 3′-end counts were merged together by RNA position and used for calculation of ScoreMEAN (derived from MAX Score described previously), as well as Scores A and B (64) and MethScore (65). Scores were calculated for two neighboring nucleotides. Profiles of RNA cleavage at selected (candidate and previously known) positions were extracted and visually inspected.

### Northern blotting

For northern blotting analysis of tRNA, 10 μg of total RNA from adult flies were resolved on 15% urea-polyacrylamide gels, transferred to Hybond-NX membrane (GE Healthcare) and EDC-crosslinked (Sigma Aldrich). The membranes were probed with 5′-^32^P end-labeled DNA oligonucleotides using T4 polynucleotide kinase (Fermentas). Hybridization was performed overnight at 38–40°C in PerfectHyb Plus (Sigma) hybridization buffer. Probe sequences are available in the Primers and Probes section. More details on NB procedure are available in (66,67).

### RNA interference in S2R+ cells

Double-stranded RNAs (dsRNA) were synthesized by *in vitro* transcription (MEGAscript^®^ T7 Kit, Ambion) of PCR products amplified from *w^1118^* genomic DNA using primers flanked by T7 promoters. Sequences of amplicon templates for dsRNA production are available from the *Drosophila* RNAi Screening Center (http://www.flyrnai.org/cgi-bin/RNAi_gene_lookup_public.pl, e.g. Ago2: DRSC10847, CG7009: DRSC39198). PCR products for T7 transcription of *fushi tarazu* (Ftz) and Firefly luciferase dsRNAs were amplified using primers: T7_Ftz_FW and T7_Ftz_Rev and T7_F_Luc_FW and T7_F_Luc_Rev, respectively (Primers and Probes section and (66)).

### S2R+ cell transfection

100 μl of cells at 10^6^ cells/ml resuspended in Schneider's *Drosophila* medium (GIBCO, Invitrogen) were plated in 96-well plates. Cells were transfected with dsRNA or co-transfected with dsRNA and the corresponding sensor using Effectene (QIAGEN) following the manufacturer's instructions. Thirty minutes after transfection 50 μl Schneider's *Drosophila* medium (GIBCO, Invitrogen), completed with 10% heat-inactivated fetal calf serum, 100 U/ml penicillin and 100 mg/ml streptomycin were added. Cells were grown at 23°C without CO_2_. After 24–48 h, CuSO_4_ was added to a final concentration of 600 μM and GFP fluorescence was followed using an inverted epifluorescence basic microscope. For Ago2-mediated miRNA pathway involvement (*automiG*), cells were co-transfected with 0.1 μg of *automiG*-vector and 0.32 μg of dsRNA targeting either *Ago2, CG7009* or *Ftz, Dcr1, Dcr2, Drosha, Ago1*. Forty eight hours later, the *automiG* promoter was induced by adding CuSO_4_ to a final concentration of 600 μM (more details in (66)).

For the luciferase assay experiment, S2R+ cells were treated for 4 days with dsRNA inactivating specifically the indicated genes. Cells were co-transfected with two plasmids expressing the Firefly and Renilla luciferases in addition to a dsRNA against Firefly. Luciferases activities were measured 48 h after transfection. The averages of the activity ratios from Firefly/Renilla luciferases from three independent biological replicates were plotted normalized to the average of a control dsRNA (GFP) which was set to 1 (+/– the standard deviations). * indicates *P* < 0.05 in a Student's *t*-test.

### Western blotting

Expression of GFP was analyzed in *automiG* induced cells by western blotting using mouse anti-GFP (Roche^®^) and anti-Mbf1 antibodies (66) as loading and transfer control. Seventy two hours after the dsRNA transfection and *automiG* vector induction, the culture medium was removed and 80 μl of Sample Buffer Laemmli 2X (Sigma^®^) was added in each well. The samples were boiled (95°C) and 18 μl were loaded onto a 4–20% Mini-PROTEAN^®^TGX™ 12 well-gel (Bio-Rad). After transfer onto a PVDF (Amersham Hybond, GE Healthcare) or nitrocellulose membrane, membranes were blocked in 5% milk, dissolved in 1× TBS-T (20 mM Tris-Base, 150 mM NaCl, Tween-20 (Polyoxyethylene sorbitane monolaureate) to 0.05%) and incubated overnight with anti-GFP (1:2000) or anti-Mbf1 (1:10.000) antibodies diluted in the blocking solution. After three times 15 min washes, appropriate secondary antibody (1:10 000) coupled to alkaline phosphatase (Promega) was added and incubated for one hour at room temperature. Detection was performed using BCIP (5-bromo-4-chloro-3-indolyl-phosphate) and NBT (nitro-blue-tetrazolium, (ThermoFischer) reagents diluted in AP buffer (100 mM Tris–HCl pH 9.5, 100 mM NaCl, 5 mM MgCl_2_).

### automiW

Experiments with the *automiW* eyes sensor were performed as described in (68). Eye images of the same aged flies were acquired with an Axio-ApoTome (Zeiss) and ZEN2 software or with a WILD M3Z (Leica) binocular combined with a Q IMAGING Color 12 bit (Q27959) camera and QCapture Pro software.

### DCV injection

Flies with the following genotypes were subjected to intra-thoracic injection with the *Drosophila C* virus (DCV): *CG7009^e02001^*/+ (controls). *CG7009^e02001^*/*CG7009^e02001^*(*CG7009* mutants). *CG5220^K28A^*/*CG5220^K28A^*(*CG5220* catalytically dead mutant). Two to four days old flies were divided in tubes of 10 (5 males + 5 females) and 20 flies from each genotype were injected with DCV while 20 other flies were injected with adult Injection Buffer (controls), containing 10 mM Tris pH 6.3 and 1 mM MgCl_2_. Each fly was injected with 4.6 nl of *DCV* concentration of 2 × 10^6^ PFU/ml (9.2 PFU/injection). Intra-thoracic injections were made using the Drummond Automatic Nanoliter Injector ‘NANOJECT II’. After injection, flies were kept at 25°C. Three to four days after the injection and prior to death, three injected flies from each genotypes and conditions (+ or – DCV) were frozen at –20°C. Two to three flies from each condition were then crushed with a pestle in TRI-Reagent (Sigma Aldrich) and total RNA was extracted as described above. DNase digestion and RT-qPCR were carried out as described with DCV_FW and DCV_Rev-specific primers and primers for Rp49 for normalization (Primers and Probes section).

### CRISPR/Cas9-mediated genome editing and genotyping

Mutant alleles for *CG5220* were generated using CRISPR/Cas9-mediated editing in *Drosophila* as previously described (69). The *CG5220*^K>A^ allele was obtained using the gRNA (guide RNA) sequence: 5′-CTTCGAGCAACTTGAAGGCACTCC-3′ and a single-stranded DNA donor template ssDNA: 5′-TTCATATATTTATTTACAATGGGGAAAACATCAAAGGACAAAAGAGATATCTATTACCGACAAGCCAAAGACGAAGGCTGGAGGGCGAGGAGTGCCTTCGCGTTGCTCCACGTGGACGAAGCCTACGGAATTCTAA-3′ for homology-dependent repair to obtain the K to A mutation in CG5220:

A mix of 150 ng/μl vector expressing the gRNA and Cas9 protein as well as 150 ng/μl ssDNA were injected in pre-blastoderm *w^1118^* embryos. Screening for flies containing the substitution was carried out by PCR on genomic DNA from single F1 males derived from crossing of injected individuals with a balancer stock. The screened sequences correspond to genomic fragments covering 438 bp of the *CG5220* gene. Fly stock denominations are *CG5220^K>A^*. Visual sequence analysis was carried out using *4Peaks* and *ApE* software.

### RT-qPCR

RNA was extracted from whole flies or from dissected ovaries using TRI-Reagent (Sigma Aldrich). After DNase digestion of total RNA using the TURBO DNA-free™ Kit (Ambion), 500 ng were used in a reverse transcription reaction with Random Primers (Promega) and SuperScript^®^ II Reverse Transcriptase (Invitrogen). The cDNA was used to perform qPCR on a CFX96 Touch™ Real-Time PCR Detection System (Bio Rad) using target-specific primers. Rp49 was used for normalization (Primers and Probes section). The analysis was performed using ΔΔ Ct, on three biological replicates. Statistical analysis using a Student's t-test was performed and *P*-values were determined.

### Production and affinity purification of recombinant fusion proteins

Glutathione *S*-transferase (GST) fusion constructs were generated by PCR amplification of full-length cDNAs of CG7009 available from BDGP (#SD16956) using standard PCR with VENT polymerase (New England BioLabs). Products were cloned between the EcoRI and NotI restriction sites of the pGEX-4T1 (GE Healthcare) vector using primers CG7009_EcoRI_ATG and CG7009_NotI_Stop. Amplification of full-length cDNAs of *CG33172* (clone MIP10235 in BDGP) was performed using standard PCR techniques using Q5 high fidelity DNA Polymerase (New England BioLabs). Amplification products were cloned between the HindIII and NotI restriction sites of the pET-28a (Novagen) vector (modified to contain FLAG peptide) using the primers CG33172_Hind_ATG and CG33172_NotI_Stop. Competent bacteria TOP10 (Invitrogen) were transformed by heat-shock with 100–200 ng of plasmid DNA according to each manufacturer's instructions. After expression on the corresponding antibiotic resistance genes by incubation for 0.5-1.5 h at 37°C under agitation of 250 rpm, 1/10 and 9/10 of the transformed bacteria were plated on LB agar plates, supplemented with the corresponding antibiotics. GST fusion proteins were expressed in *Escherichia coli* BL21 Star (DE3) (Invitrogen) or C41 (70) and purified over glutathione-coupled resin (Pharmacia) as previously described (71,72). The same protocol was used for purification of pET-28a Flag fusion proteins. Bound peptides were eluted with 400 μg/ml Flag peptide (Sigma) in BC100 buffer for 20 min on ice.

### *In vitro* interaction of GST-CG7009 and FLAG-CG33172

Briefly, GST- alone (control) or fusion proteins GST::G7009 (pGEX4T1-CG7009) and FLAG::CG33172 (pET28a-FLAG-CG33172) were co-expressed in C41 (70) bacteria and purified over Flag-coupled resin (Sigma). Bound proteins were washed three times in 500 mM KCl and eluted on Bio-spin disposable chromatography columns (Bio-Rad) with flag peptide as described in (72). Western blot of the immunoprecipitated recombinant proteins was performed as described in the above section using anti-GST HRP (horseradish peroxidase) conjugate (1:10 000 Amersham GE Healthcare) for 60 min at room temperature under agitation. HRP was detected by enhanced chemiluminescent (ECL).

### RNA-seq on S2R+ cells

Knock downs for *CG7009* and *LacZ* (control KD) in S2R+ were performed in a serum-free medium using 7.5 μg of dsRNA per 10^6^ cells and stopped 2 h after cell starvation with the addition of the serum-supplemented medium. dsRNA treatment was repeated after 48 h. Cells were collected and total RNAs were extracted 96 h after the first treatment. Libraries were prepared using the Illumina TruSeq Sequencing Kit by following the manufacturer's protocol for paired-end read mode and directional sequencing on an Illumina NextSeq 500 with a read length of 42 bp.

### Computational analysis of S2R+ RNA-seq experiments

Basecalling and demultiplexing were performed using bcl2fastq (v2.19). Individual samples were mapped using STAR ((73), v2.5.2b) against ensembl release 90 of the *D. melanogaster* genome (BDGP6). Gene counts were derived using featureCounts ((74), v. 1.5.1). RNA-Seq Analysis Differential expression analysis was performed using Bioconductor v2.38/ DESeq2 v1.18.1 (75,76). Genes were called sig. diff. expressed with an FDR below 5%. The sample Ctrl_3 was excluded as an outlier from the differential expression analysis. The gene list deduced from this analysis is available online at NAR as supplementary material (excel file) and the corresponding libraries publicly accessible for download (see Data availability for detail).

### Small RNA sequencing and computational analysis

Library preparation was performed at Fasteris (http://www.fasteris.com). Total RNAs from ovaries were size selected (18 to 30 nt) on denaturing PAGE. The small RNA fraction was then depleted from the 2S rRNA (30 nt) using a highly specific probe developed by Fasteris. 2S rRNA-depleted small RNAs were used to generate multiplexed libraries with Illumina TruSeq Small RNA library preparation kits. Libraries were sequenced using Illumina HiSeq 4000 platforms. Fastq sequence reads were trimmed of the adapter sequences (5′-TGGAATTCTCGGGTGCCAAG-3′) and reads were mapped using Bowtie (77). Only 19–29 nt reads matching the reference sequences with zero or one mismatches were retained for subsequent analyses. For global annotation of the libraries, we used the release 6 of fasta reference files available in FlyBase, including transposon sequences (dmel-all-transposon_6.fasta) and the release 20 of miRBase (http://www.mirbase.org/). For library comparisons, read counts were normalized to the total number of small RNAs matching the *D. melanogaster* genome (release 6). Sequence length distributions and small RNA mappings were generated from bowtie alignments using Python and R (http://www.r-project.org/) scripts, which were wrapped and run in *Galaxy* instance from ARTbio platform (http://artbio.fr/). Tools and workflows used in this study are available for download at NAR as supplementary material (.ga files). The small RNA sequencing data discussed in this publication are accessible at European Nucleotide Archive (ENA) at EMBL-EBI under accession number PRJEB35301 (https://www.ebi.ac.uk/ena/data/view/PRJEB35301).

### β-Gal staining of dissected ovaries

Stainings of ovaries were performed as follows: specific sensor lines were crossed with respective RNAi lines for knockdown in germ cells (*nanos*-GAL4) or follicle cells (*tj*-GAL4) of the G1 female ovaries followed by β-Gal staining performed on 3–7 days-old females. Ovaries were dissected in cold 1× PBS, kept on ice, fixed in glutaraldehyde (0.2%)/formaldehyde (2%)/1× PBS at room temperature for 5 min followed by three washes in 1× PBS. Tissues were then incubated in freshly prepared staining solution (1× PBS pH 7.5, 1 mM MgCl_2_, 4 mM potassium ferricyanide, 4 mM potassium ferrocyanide, 1% Triton X-100, 2.7 mg/ml X-Gal) at 25°C overnight for *Gypsy::LacZ* detection and for 90 min for *burdock::LacZ* detection. The staining solution was prepared with 8% X-Gal (as dimethylformamide solution). After staining, tissues were transferred into 50% glycerol/50% EtOH and mounted for imaging.

### Imaging

Ovary and eye images were acquired with a WILD M3Z (Leica) binocular combined with a Q IMAGING Color 12 bit (Q27959) camera and QCapture Pro software. Ovary sizes (area) were calculated in pixels using ImageJ. An unpaired Mann–Whitney (Wilcoxon) test was performed to evaluate the significance of the area differences (*P-*value < 0.05) between mutant ovaries (*n* = 11) and genetic rescue ovaries (*n* = 10).

### Weighing

Average weight for flies in milligrams (mg) was calculated for flies (3 days after hatching) measured on a precision balance (≥0.001 g) after heat dehydration at 95°C (15 min in an open 1.5 ml eppendorf tube) of frozen bodies (n ≥ 14, where each *n* is a batch of 10 flies). *P*-value < 0.001 in a Student's *t*-test.

### Lifespan assays

2–3-day-old male flies Da-GS; UAS-RNAi *CG5220*, *CG7009* were kept at 25 °C in vials with standard medium complemented (RU200) or not (RU0) with RU486. RNAi transgene induction using the Da-Gal4-GS (GeneSwitch) lines was described in (78). Briefly, the Gal4-GS protein is a GAL4 modified protein that recognizes and activates UAS-dependent transgenes only in the presence of RU486 added into *Drosophila* medium food. The number of flies tested was five times 30 flies. To monitor survival rates over time, flies were counted and transferred into new tubes every 2–3 days. Constitutive expression of *CG5220*, *CG7009* KD transgenes was induced by RU486 exposure (20 mg/ml) during adulthood. The exact same protocol was followed for the genetic mutants with the exception of the RU486 exposure. The number of flies tested was five times 30 flies per genotype.

### Climbing assays

Sixteen day-old flies were gender-separated and placed into measuring cylinders to assess their locomotion using the climbing assay reported previously (79). Briefly, flies were tapped to the bottom and the number of flies that climb over the 10 cm threshold in 10 s intervals were recorded and counted. Ten female or male flies were used per experiment. Shown data are an average of six independent experiments. *P < 0.01; ***P < 0.0001 in a Student's t-test.

### Drosophila stocks

**Table:** 

Lab stock ID#	Category	Genotype	Notes
w1118	Mutant allele	w1118	FlyBase ID FBal0018186
CG7009^e02001^	Mutant allele	w1118; CG7009e02001 (mini-white)	Bloomington stock #18008 cleaned by backcrossing over 10 generations.
Def9487	Deficiency for *CG7009*	w1118; Df(3R)ED10845, P{3′.RS5+3.3′}ED10845 / TM6C, cu1 Sb1	Bloomington stock #9487
Def3340	Deficiency for *CG7009*	Df(3R)e-R1, Ki1/TM3, Sb1 Ser1	Bloomington stock #3340
BAC	Rescue allele for CG7009	w1118; CH322 177K12 (Pacman)	This work, FlyBase cloneFBlc0000784
CG5220^K>A^	Mutant allele	w1118; CG5220 248.5.2 K28A/ TM3, Ser	This work
CG7009^e02001^, CG5220 ^K>A^	Double mutant allele	w1118; CG7009e02001-G10 (mini w), CG5220 248.5.2 K28A/ TM6, Tb, Sb	This work
GMR-GAL4	GAL4 driver	P{GMR-GAL4.w-}	FBti0072862
tj-GAL4	GAL4 driver	P{tj-GAL4.U}	FBtp0089190
IR-white	RNAi white	y1 v1; P{TRiP.HMS00017}attP2	Bloomington stock: 33623
IR-Piwi	RNAi Piwi	w1118; UAS-IR(Piwi) CG 6122	VDRC N° stock: 22235 (GD)
IR-Ago2	RNAi Ago2	w1118; UAS-RNAi(Ago2)	VDRC N° stock: 49473 (GD)
IR-Ago2	RNAi Ago2	w1118;; UAS-RNAi(Ago2)	VDRC N° stock: 100356 (KK)
IR-CG7009	RNAi CG7009	w1118;; UAS-RNAi (CG7009)	VDRC N° stock: 27789 (GD)
IR-CG5220	RNAi CG5220	w1118;; UAS-RNAi (CG5220)	VDRC N° stock: 34972 (GD)
IR-CG5220	RNAi CG5220	w1118;; UAS-RNAi (CG5220)	VDRC N° stock: 108672 (KK)
IR-CG33172	RNAi CG33172	w1118; P{KK102903}VIE-260B	VDRC N° stock: 100006 (KK)
IR-CG15618	RNAi CG15618	w1118;; UAS-RNAi (CG15618)	VDRC N° stock: 40006 (KK)
shRNA-Moon	shRNA Moonshiner	w;; pW20>moon_sh2[attP2]/TM3, Sb	(Andersen *et al.*, 2017)
automiW	Genetic sensor	UAS-automiW (w+)	(Besnard-Guerin *et al.*, 2015)
Gypsy LacZ	Genetic sensor	R; tjgal4 / Cyo; Gypsy LacZ / Tb,Sb	(Sarot *et al.*, 2004)
Burdock LacZ	Genetic sensor	UAS>Dcr2; nosNGT-Gal4; nos-NLS::eGFP::LacZ::Burdock-3′UTR	(Handler *et al.*, 2013)
CC2	Double balanced line	w*; T(2;3)ap[Xa] / CyO, P{[w*] = Act-GFP}CC2; TM6C, Sb1 Tb1	Home-made
Da-GS	Inducible Gal4 under Daughterless promotor	w1118; DaGS-45	(Tricoire *et al.*, 2009)

### Primers and probes

**Table:** 

Primer	Sequence	Experiment
SD22 (RP49_FW)	GACGCTTCAAGGGACAGTATCTG	RT-qPCR
SD23 (RP49_Rev)	AAACGCGGTTCTGCATGAG	
CG7009_qPCR2_FW	GAGTTTTGTCTGCCCGATGG	
CG7009_qPCR2_Rev	ACTTGGCTCGTTTTCTGCAG	
CG5220_qPCR2_FW	GATTAACCCTGCTCGCGATG	
CG5220_qPCR2_Rev	TCCAGGGGATAAGATGCGTC	
DCV_FW	TCATCGGTATGCACATTGCT	
DCV_Rev	CGCATAACCATGCTCTTCTG	
(Gypsy (2772) _FW	CCAGGTCGGGCTGTTATAGG	
(Gypsy (2663) _Rev	GAACCGGTGTACTCAAGAGC	
LacZ_2_FW	ACTATCCCGACCGCCTTACT	
LacZ_2_Rev	GTGGGCCATAATTCAATTCG	
Roo_Fw	CGTCTGCAATGTACTGGCTCT	
Roo_Rev	CGGCACTCCACTAACTTCTCC	
Invader1_Fw	GTACCGTTTTTGAGCCCGTA	
Invader1_Rev	GCGAAGTAGCCTCCTTGATG	
R2_Fw	TAGCCCCGTAGAATGCCATT	
R2_Rev	AGTGGTTTCCTTTCCCTCGA	
Ago2_Fw	AGTGTAATAATCAGACGATTGG	
Ago2_Rev	AGGGATGGGTCACATCGGCTCC	
CG7009_EcoRI_ATG	AAGAATTCATGGGCAGGACTTCGAAGGATA	Cloning of recombinant proteins
CG7009_NotI_Stop	GCAGCGGCCGCTTACGTTACACAGGCACCTAACT	
CG33172_Hind_ATG	CAACTGGCAAAGCTTATGGTTTTGATTTCTGACGC	
CG33172_NotI_Stop	ACTGGCAGCGGCCGCTTAAAGTATATTACTTATGCTCATAGTCTGC	
T7_Ftz_FW	GAATTGTAATACGACTCACTATAGGGCTGGCAAAGTCGCCATTCT	dsRNA synthesis
T7_Ftz_Rev	GAATTGTAATACGACTC-ACTATAGGGCCAACATGTATCACCCCCA	
T7_F_Luc_Fw	TAATACGACTCACTATAGGGATGCACATATCGAGGTGGAC	
T7_F_Luc_Rev	TAATACGACTCACTATAGGGAGAATCTCACGCAGGCAGTTC	
CG7009 T7 F	ttaatacgactcactatagggagaTCCGATCGAAGGAGTCAAAC	
CG7009 T7 R	ttaatacgactcactatagggagaGCCATTTCTTCAACATTTCCTC	
LacZ T7 F	ttaatacgactcactatagggagaCAGGCTTTCTTTCACAGATG	
LacZ T7 R	ttaatacgactcactatagggagaCTGATGTTGAACTGGAAGTC	
CG7009-dTOPO FW	CACCATGGGCAGGACTTCGAAGGAT	Genotyping
CG7009-dTOPO Rev	TTACGTTACACAGGCACCTAACTTC	
CG7009-middle FW	TCCACTGGAATGCACGACTT	
CG7009-middle Rev	AAGTCGTGCATTCCAGTGGA	
pB-3SEQ	CGATAAAACACATGCGTCAATT	
pB-5SEQ	CGCGCTATTTAGAAAGAGAGA G	
VIE0197: 5220 mutant screening FW	GATATATCGATAGGCTGGCC	
VIE0198: 5220 mutant screening Rev	CAGGTATCGTAGAGTTTCCG	
tRNA Phe GAA 5′ probe (MA_075)	GCTCTCCCAACTGAGCTATTTCGGC	Northern blot
5S-rRNA probe (CA primer 5399)	CAACACGCGGTGTTCCCAAGCCG	
AS-miG1	AGAACGGCATCAAGGTGAACTTC	
2S-rRNA	TGCTTGGACTACATATGGTTGAGGGTTGTA	
Bantam	AATCAGCTTTCAAAATGATCTCA	
esi-2.1	GGAGCGAACTTGTTGGAGTCAA	

 

## RESULTS

### An RNAi screen identifies CG7009 as regulator of small RNA-mediated silencing pathways

**Figure 1.**
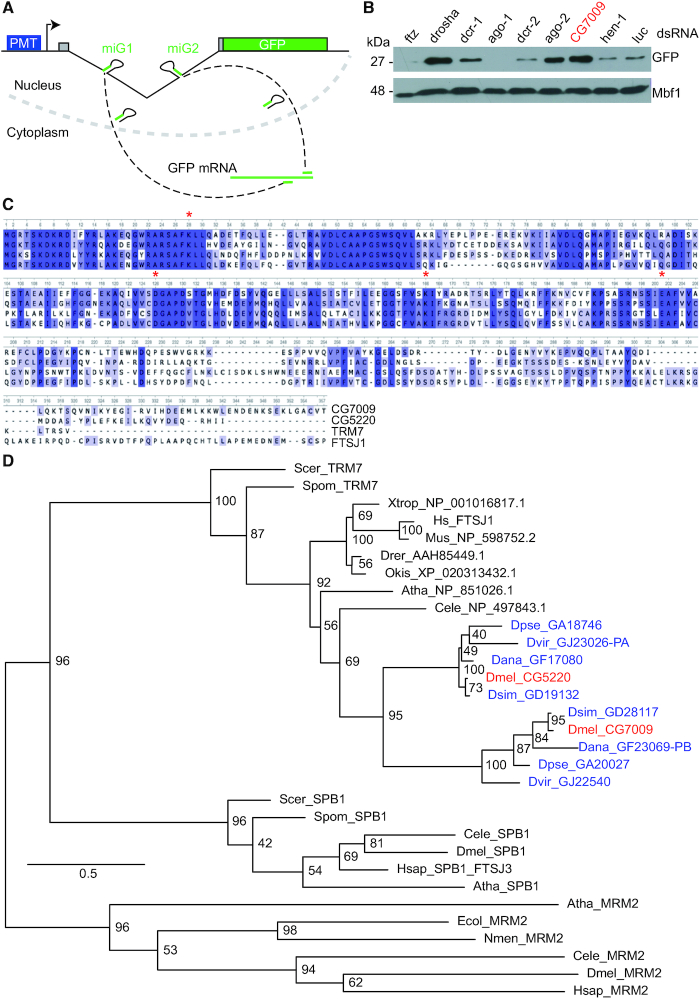
Identification of CG7009 and CG5220 as conserved TRM7 family proteins. (**A**) The *automiG* sensor. *automiG* carries a copper-inducible promoter (PMT) that drives the expression of two miRNAs (miG1 and miG2) and the GFP mRNA. Both miRNAs target the GFP mRNA with perfect complementarity. *AutomiG* repression is dependent on Ago2, Drosha, Pasha, Dicer-1 and Dicer-2 functions as reported previously (66). (**B**) CG7009 function affects *automiG* repression. Cells were soaked with the indicated double-stranded RNA (dsRNA), followed by *automiG* induction using copper sulfate. GFP expression was analyzed by western blotting. The Mbf1 protein was used as loading control. dsRNA against *Fushi tarazu* (*Ftz*) and luciferase (*luc*) served as negative KD controls. kDa: kilo Dalton. (**C**) Multiple amino acid sequence alignment of CG7009, CG5220, TRM7 (*S. cerevisiae*), and FTSJ1. The conserved predicted catalytic tetrad amino acids K–D–K–E are marked by red asterisks. Dark blue points to conserved amino acid in the three organisms. (**D**) *Drosophila* species evolved two TRM7 family proteins. Phylogenetic analysis of TRM7 and SBP1 MTases. The SBP1 family member RrmJ acting on rRNA was used as an outgroup. Color blue indicates TRM7 family proteins in *Drosophila* species other than *D. melanogaster*. Red indicates TRM7 family proteins in *D. melanogaster*.

We previously developed and characterized a self-silencing genetic sensor (*automiG*) that combines the expression of GFP with two miRNAs, miG-1 and miG-2, targeting GFP mRNA (Figure 1A and (66)). *AutomiG* self-silencing reports on the activity of canonical miRNA biogenesis factors such as Drosha and Dicer1 (Dcr1), and the function of siRNA-induced silencing complex (siRISC) factors such as Argonaute2 (Ago2) and Dicer2 (Dcr2) (66). Impairing the function of miRNA biogenesis or Ago2 silencing activity thus causes the de-repression of *automiG* self-silencing resulting in the expression of GFP. To identify additional regulators of these two RNA silencing pathways, a genome-wide RNA interference screen was performed in *Drosophila* S2 cells expressing the *automiG* sensor. Using a double-stranded RNA (dsRNA) collection library (the DRSC 2.0) allowed the down-regulation of 94.3% of all annotated *Drosophila* genes. The screen identified known regulators of miRNA biogenesis such as Drosha and Pasha, as well as siRNA pathway silencing key actors like Ago2 and Droj2 (80), demonstrating the validity of this approach. In addition, we identified 17 genes affecting a*utomiG* silencing, which had not yet been reported to act in siRNA and/or miRNA pathways (Supplementary Figure S1A). Among those, *CG7009* stood out as an uncharacterized gene with sequence identity to annotated Nm-MTases. RNAi-mediated inactivation of CG7009 in S2 cells expressing *automiG* resulted in increased GFP expression when compared to control constructs (Figure 1B), as well as in a decrease of Ago2-loaded miG1, but not Ago1-loaded bantam miRNAs (Supplementary Figure S1B). In addition, a dual luciferase assay reporting specifically on siRNA pathway activity in S2 cells (81,82) confirmed that Dcr2/Ago2-dependent silencing was affected in cells with down-regulated CG7009 expression (Supplementary Figure S1C).

In order to obtain insights into the impact of CG7009 loss on gene expression control through Dcr2/Ago2-mediated post-transcriptional gene silencing, we performed a transcriptome analysis in *Drosophila* S2 cells upon knockdown (KD) of CG7009 expression. Surprisingly, KD of CG7009 led to the deregulation of only 110 genes (FDR < 0.01). Strikingly, the most statistically significant de-regulated gene (40% decrease, log_2_FC –0.7, FDR-adjusted *P*-value 7.73e^−118^) were *Ago2* transcripts (Supplementary Figure S1D), suggesting that CG7009 may act upstream of the siRNA pathway by regulating *Ago2* mRNA levels. The downregulation of *Ago2* transcripts in *Drosophila* S2 cells KD for CG7009 expression was confirmed by RT-qPCR on four independent biological replicates (Supplementary Figure S1E).

This genetic screen using *automiG* thus identified CG7009, a potential Nm-MTase, as a factor involved in miRNA biogenesis and/or Dcr2/Ago2-mediated post-transcriptional gene silencing.

### *CG7009* encodes a predicted Nm-MTase

Amino acid (aa) sequence analysis suggested that the protein encoded by CG7009 in *D. melanogaster* harbours a methyltransferase domain belonging to the conserved RlmE family and TRM7 subfamily of the class I-like SAM-binding methyltransferase superfamily (48). Sequence alignment of the putative CG7009 protein with the yeast Nm-MTase TRM7 showed 52% aa sequence identity, including the conserved KDKE motif in the active site, with 66% aa coverage (Figure 1C). FTSJ1 is the human ortholog of TRM7 (49). CG7009 shares 51% aa identity and 86% aa coverage with FTSJ1 (Figure 1C). Surprisingly, further alignment of CG7009 protein sequence with proteomes of different *Drosophila* species uncovered an additional gene, *CG5220*, whose annotated protein in *Drosophila melanogaster* displays 63% aa sequence identity with CG7009 (Figures 1C and D). Like CG7009, CG5220 was an uncharacterized protein with an amino acid composition that clearly showed an Nm-MTase signature (Figure 1C). Importantly, it was previously reported that overexpression of *Drosophila* CG5220 rescued the growth phenotype observed in *trm7Δ* mutant yeast (45). As CG7009, CG5220 displays high sequence similarity to TRM7 (48% aa identity and 83% aa coverage) as well as to FTSJ1 (58% aa identity and 82% aa coverage, Figure 1C). These findings pointed to CG7009 and CG5220 as potential paralogs and conserved members of the TRM7 Nm-MTases family in *Drosophila*.

### Mutations in *CG7009* or *CG5220* are viable and fertile

**Figure 2.**
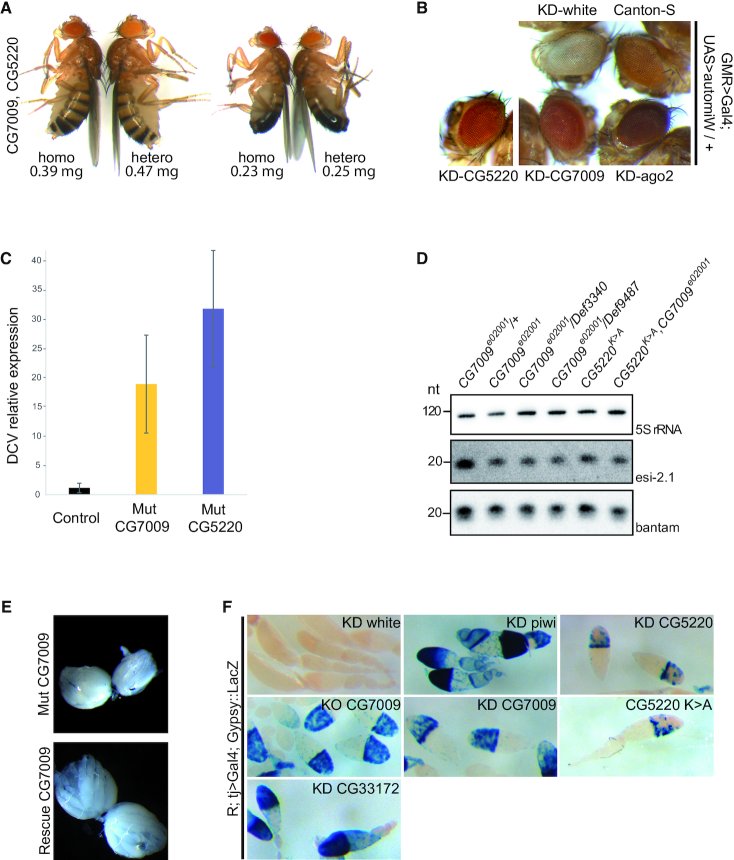
CG7009 and CG5220 affect small RNA silencing pathways. (**A**) Homozygous *CG7009, CG5220* double mutant flies display reduced adult weight and size. Images of adult females and males *CG7009, CG5220* homozygous double mutants (homo) compared to heterozygous double mutants (hetero). Below the images the average weight for flies in milligrams (mg) calculated for 3 days-old flies measured on a precision balance (*n* > 100 flies/ genotype; *P*-value < 0.001 in a Student's *t*-test) is indicated. The percentage change for female heterozygous *CG7009* and *CG5220* mutants *versus* homozygous *CG7009*, *CG5220* double mutant represents a decrease of 20.5%. The percentage change for male heterozygous *CG7009*, *CG5220* double mutant versus homozygous *CG7009*, *CG5220* double mutant represents a decrease of 8%. (**B**) CG7009 and CG5220 modulate Ago2-dependent gene silencing in somatic tissues. The UAS>*automiW* construct is a sensor derived from *automiG* by which two miRNAs target the *white* gene (68). KD indicates eye-specific GMR-Gal4/UAS-RNAi-mediated inactivation of the respective genes (*white*, *CG7009*, *CG5220* or *Ago2*). Canton-S *was* used as control for eye color determination. Darker eye coloration than Canton-S (top right) indicates that the *white* gene is not inactivated by Ago2-loaded miRNAs targeting *white*. Images were taken at the same age (5 days after hatching) for different genotypes. (**C**) The siRNA-dependent viral defence is compromised in *CG7009* and *CG5220* mutants. RT-qPCR using *Drosophila* C Virus (*DCV*)-specific primers three days after injection with *DCV* solution or solution free of DCV as control (not shown) in heterozygous *CG7009^e02001^* mutants (Control) or homozygous *CG7009^e02001^* (Mut CG7009) and *CG5220^K>A^* (Mut CG5220) mutants. Shown is *DCV* expression relative to *Rp49*. Error bars represent the standard deviation (s.d.) of the mean between two (*n* = 2) biological replicates (*n* is a mix of 2–3 flies). (**D**) Endogenous siRNA (esi-2.1) expression is reduced in *CG7009* and *CG5220* mutants. Northern blotting on total RNAs extracted from adult flies of the indicated genotypes was performed using esi-2.1, bantam-specific probes and a 5S rRNA probe as loading and transfer control. nt: nucleotide. (**E**) The *CG7009* mutation is associated with ovarian size reduction. The images show representative examples of ovaries from 4 days-old fertilized females raised on fresh yeast from trans-heterozygous *CG7009^e02001^/Def9487* mutants (Mut CG7009) and Rescue *CG7009 (BAC)/ +; CG7009^e02001^/Def9487* mutants (Rescue CG7009); *n* ≥ 10 for each genotype; Mut: mutant. An unpaired Mann–Whitney (Wilcoxon) test was used to calculate the significance of the ovary area differences between mutant and rescue genotypes. The percentage change from the mutant and the rescue genotypes represents a decrease of 10.5% with a *P-*value < 0.05 (W = 23, *P*-value = 0.02416). (**F**)CG7009 and CG5220 are involved in *gypsy* TE-repression in *Drosophila* ovaries. The *Gypsy::LacZ* sensor is silenced through *tj*>Gal4-mediated expression of an UAS-RNAi line (KD) against the *white* gene in follicle cells (R; *tj*>Gal4/ +; Gypsy::LacZ/UAS-*white*-RNAi). *Gypsy* silencing is disrupted using RNAi lines against *Piwi* (KD *piwi*), *CG7009* (KD CG7009), *CG5220* (KD CG5220) and *CG33172* (KD CG33172). The *Gypsy::LacZ* sensor was also de-repressed in *CG7009* null mutants (KO CG7009) and *CG5220*^K>A^ homozygous mutants (CG5220 K>A). KD: knock down; KO: knock out; no blue coloration = no β-Gal staining.

To investigate the function of CG7009 and CG5220 during *Drosophila* development and to characterize the potential enzymatic activity of their gene products, we characterized existing mutations in *CG7009*, but also generated *CG5220* mutant flies. For *CG7009*, one *piggyBac* transposon insertion line (*CG7009^e02001^*) and two genomic deletion lines (*Def3340* and *Def9487*) were obtained and precisely mapped at the molecular level (Supplementary Figure S2A–E). Both *CG7009^e02001^* homozygous mutants and trans-heteroallelic combinations with both deficiencies were incapable of transcribing *CG7009* properly. In addition, a transgenic rescue line containing the *CG7009* genomic locus was established through BAC transgenesis (83) resulting in an insertion of ∼20 kb genomic sequence in an ectopic genomic location (Supplementary Figure S2D, E). To address the function of CG5220, CRISPR/Cas9-mediated genome editing was used to create a *CG5220* mutant allele (*CG5220^K>A^*), which substituted a conserved lysine at position 28 in the predicted catalytic domain with alanine (Figure 1C and Supplementary Figure S2F). The same substitution was reported to abolish the catalytic function of both yeast TRM7 and human FTSJ1 (49). Flies homozygous for either *CG7009^e02001^* or *CG5220^K>A^* or trans-heterozygous *CG7009* mutants, as well as *CG7009^e02001^*, *CG5220^K>A^*double mutants survived until adulthood under standard conditions. We observed neither a major growth defect as reported for yeast (46,48) nor significant developmental delays in flies homozygous for either *CG7009^e02001^* or *CG5220^K>A^* or trans-heterozygous *CG7009* mutants. However, flies that were *CG7009^e02001^*,*CG5220^K>A^*double mutant showed a measurable reduction of size and weight when compared to controls (Figure 2A).

### CG7009 and CG5220 contribute to efficient miRNA Ago2-mediated RNA silencing *in vivo*

To address whether CG7009 affected small RNA silencing pathways *in vivo*, we expressed the *automiW* sensor, which is based on the knockdown of the *white* gene in the developing eye by means of *white*-targeting miRNAs loaded into Ago2 (68). Thereby, as *automiG* in cell culture, *automiW* is reporting on both miRNA biogenesis and Ago2-dependent silencing activities in flies. Combining this sensor construct with RNAi-mediated knockdown of CG7009 or CG5220, we observed increased eye coloration when compared to controls (Figure 2B). This result indicated that Ago2-dependent silencing or/and miRNAs biogenesis affecting this reporter was non-redundantly perturbed after knockdown of CG7009 or CG5220 expression, implicating thus both genes in general miRNA biogenesis and/or Ago2-dependent small interfering RNA-mediated gene silencing *in vivo* in *Drosophila*.

### siRNA-mediated RNA silencing is impaired in *CG7009* and *CG5220* mutant flies

As small interfering RNA-mediated silencing is required for viral defence in *Drosophila* (84), we tested whether viral defence was impaired in *CG7009* or *CG5220^K>A^* adult mutant flies. To this end, purified *Drosophila* C virus (*DCV*) was injected into the thorax and the viral load was monitored by qRT-PCR 4 days after infection. The results of these experiments showed that flies lacking CG7009 or CG5220 function were significantly more sensitive to *DCV* infection when compared to control flies (Figure 2C). Furthermore, these results also suggested that *CG7009* and *CG5220^K>A^* mutants failed to initiate or maintain a proper response to viral infection which, together with the results of the *automiG*, *automiW* and siRNA-activity reporter assays (Figures 1B and 2B, Supplementary Figure S1C), strongly supported that both gene products were required for efficient Ago2-dependent small interfering RNA-mediated silencing activities in *Drosophila*.

To test whether Nm-MTase mutant conditions also affected other small RNAs, northern blotting was performed for interrogating the steady state levels of *esi-2.1*, an endogenous siRNA that depends on both Ago2 and Dcr2 activities (85). The results of these experiments showed that flies lacking CG7009 or CG5220 function displayed clearly reduced *esi-2.1* levels when compared to control flies (Figure 2D).

### piRNA-mediated RNA silencing is affected in *CG7009* and *CG5220* mutant flies

During the characterization of *CG7009* mutants, we noticed that ovaries were significantly reduced in size when compared to BAC-rescued control flies (>10%; *P* < 0.05, Figure 2E). This ovarian size reduction was similar to previously described phenotype in several piRNA pathway gene mutants (86). Although the original *automiG*-based genetic screen was specifically designed to identify genes involved in miRNA biogenesis or Ago2-mediated silencing pathways, we tested whether CG7009 and CG5220 function also affected transposable element (TE) silencing through the piRNA pathway. To this end, the activity of a somatic piRNA-mediated silencing reporter (87) was monitored in adult ovaries. This reporter faithfully recapitulates the expression of the retro-transposon *gypsy* in ovarian follicle cells, in which abundant somatic piRNAs are produced in defence against mobile elements (87,88). Remarkably, piRNA-mediated silencing of this reporter was de-repressed in soma upon both, somatic follicle cell-specific knockdown of *CG5220* and *CG7009* expression, and also in *CG7009* or *CG5220^K>A^* mutants (Figure 2F). Furthermore, expression of both *LacZ* and endogenous *gypsy* mRNAs was elevated in *CG7009* mutants (Supplementary Figure S3A). In addition, the activity of a second piRNA-mediated silencing reporter in adult ovarian germ cells (89) was de-repressed upon germline-specific knockdown of CG5220 and CG7009 expression (Supplementary Figure S3B). Finally, in addition to *gypsy* and *burdock*, the expression of additional TEs (Roo, Invader1 and R2) was elevated in *CG7009* mutants (Supplementary Figure S3C). Taken together, these results suggested that both Nm-MTases contribute to piRNA pathway-mediated TE silencing in *Drosophila*.

### Small non-coding RNA biogenesis is not globally affected in *CG7009* mutants

**Figure 3.**
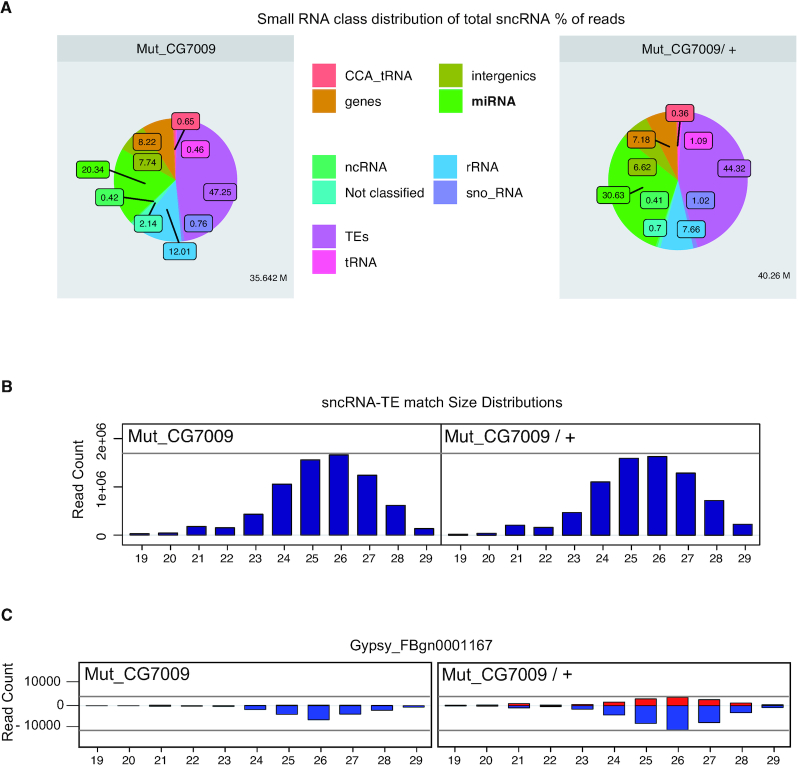
Small non-coding RNA biogenesis is not globally affected in *CG7009* mutants. (**A**) Class distribution of ovarian small RNAs (19–29 nt) matching the whole *Drosophila* genome reveals a significant decrease of miRNAs between control (Mut_CG7009/ +) and *CG7009^e02001^* homozygous mutants (Mut_CG7009). Circle circumference represents the depth of the library (indicated in million reads at the bottom right). *n* = 2 libraries for each genotype: Mut_CG7009: *CG7009^e02001^* homozygous mutant, while *CG7009^e02001^/ +* represents the control heterozygous condition. Color code in the middle panel indicates each small RNA read matching to a category of *Drosophila* small RNA (miRNA: microRNA derived sequences, rRNA: rRNA derived sequences, snoRNA: small nucleolar RNA, gene indicated small RNA sequences derived from a coding genes, etc.). Sequencing of two *CG7009^e02001^* homozygous mutant libraries (*M* = 201490, SD = 21875.26) showed significantly decreased miRNA read numbers *t*(2) = 5.89735 when compared to the two *CG7009^e02001^/+* control libraries (*M* = 307354, SD = 14248.2). The *P*-value is 0.04867. The result was significant at *P* < 0.05. (**B**) Size distribution (19–29 nt) of small RNA read counts matching TE-derived sequences in *Drosophila* ovaries. One experiment is shown for each genotype, Mut_CG7009: *CG7009^e02001^* homozygous mutant, while *CG7009^e02001^/ +* represents the control heterozygous condition. Horizontal grey line indicates the highest value and is depicted for better comparison between the two presented conditions. (**C**) Size distribution of small RNA read counts from ovaries matching *gypsy* retro-element sequences reveals that 23–29 nt piRNAs against *gypsy* are reduced in *CG7009* mutants compared to controls. Positive and negative values correspond to sense (red) and antisense (blue) reads, respectively. Horizontal grey lines indicate the highest values (sense and antisense) and are depicted for better comparison between the two presented conditions.

To gain more insights into the observed de-regulation of small non-coding RNAs in Nm-MTase mutants, we performed a small RNA sequencing analysis in ovaries from *CG7009* mutants and controls. The results showed that neither the sncRNA class distribution (Figure 3A) nor the TE-derived sncRNAs size profile distribution (Figure 3B) was globally affected in *CG7009* mutant ovaries when compared to controls.

However, when focusing the analysis on *gypsy*-derived piRNAs, we detected a decrease of both sense and antisense piRNAs targeting *gypsy* in *CG7009* mutants when compared to controls (Figure 3C), which confirmed the *gypsy* de-repression observed when using a *gypsy* sensor line and RT-qPCR assays (Figure 2F and Supplementary Figure S3A).

Notably, the entire miRNA population was significantly (*P* < 0.05) decreased in *CG7009* mutants when compared to controls (∼ 20% versus ∼30% respectively, Figure 3A) supporting the observed decrease of Ago2-loaded miRNAs (miG1) after knockdown of CG7009 expression in *automiG-*expressing S2 cells (Supplementary Figure S1B).

Taken together, these small non-coding RNA sequencing analyses suggested that the de-regulation of small RNA-mediated gene silencing observed in both *CG7009* and *CG5220* mutants (Figures 2D and F, Supplementary Figures S3A–C) was not caused by a global failure in small RNA biogenesis.

### Mutations in *CG7009* and *CG5220* affect lifespan and mobility

**Figure 4.**
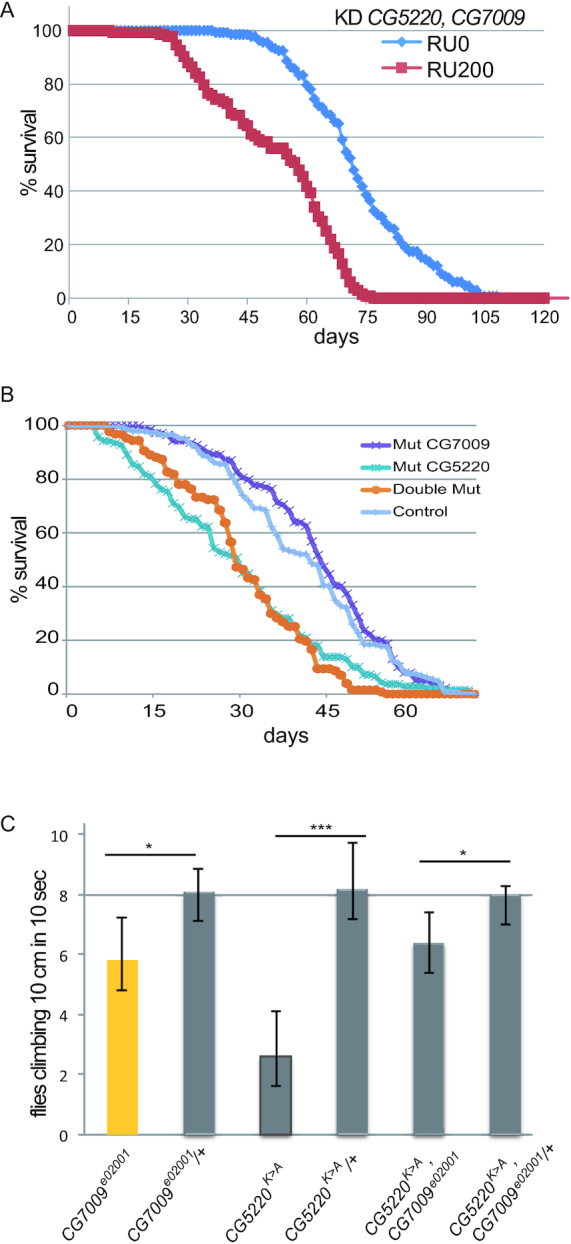
Mutations in *CG7009* and *CG5220* affect lifespan and mobility. (**A**) Simultaneous downregulation of *CG7009, CG5220* expression results in reduced lifespan. Survival curves of males expressing an RU486-inducible RNAi transgene against CG7009 and CG5220 with (RU200) or without (RU0) RU486-mediated RNAi transgene induction. Constitutive expression (RU200) of *CG5220*, *CG7009* KD transgenes was induced by RU486 exposure (20 mg/ml during adulthood). The curves represent the average values of five biological replicates of 30 flies per experiment. (**B**) Homozygous double mutant *CG5220^K>A^*, *CG7009^e02001^* results in reduced lifespan. Survival curves of indicated males homozygous mutant for *CG5220^K>A^*(Mut CG5220), homozygous mutant for *CG7009^e02001^* (Mut CG7009), homozygous double mutant *CG5220^K>A^*, *CG7009^e02001^* (Double Mut) and heterozygous *CG7009^e02001^*/+ used as control condition (Control). The curves represent the average values of five biological replicates of 30 flies per experiment. (**C**) CG7009 and CG5220 control fly behavior. Bar graphs represent data of 16 days-old male or female flies (10 flies/experiment) that climbed over 10 cm in 10 s (≥6 independent measurements for each genotype) and the standard deviation of the mean. *P < 0.01; ***P < 0.0001 in a Student's *t*-test.

Although a size and weight reduction of *CG7009*, *CG5220* double mutant adult flies could be observed (Figure 2A), no other severe mutant phenotypes affecting adult fly morphology was noticeable. Importantly however, using a drug-inducible UAS/GAL4 system (78), *CG7009* and *CG5220* double knockdown flies displayed reduced lifespan when compared to controls of the same genetic background without induction of the KD transgenes. Indeed, double knockdown flies lived, on average, ∼25 days shorter than controls (Figure 4A). The *CG5220^K>A^*, *CG7009^e02001^* double mutant flies also displayed reduced life span, confirming the effect of the KD experiments (Figure 4B). Lastly, homozygous *CG7009^e02001^* or *CG5220^K>A^*mutant flies as well as *CG7009^e02001^*,*CG5220^K>A^* double mutants appeared sluggish and less active in a climbing assay (79), supporting the notion of general locomotion defects in flies with impaired Nm-MTase function (Figure 4C).

### CG7009 and CG5220 are Nm-MTases acting on tRNAs

**Figure 5.**
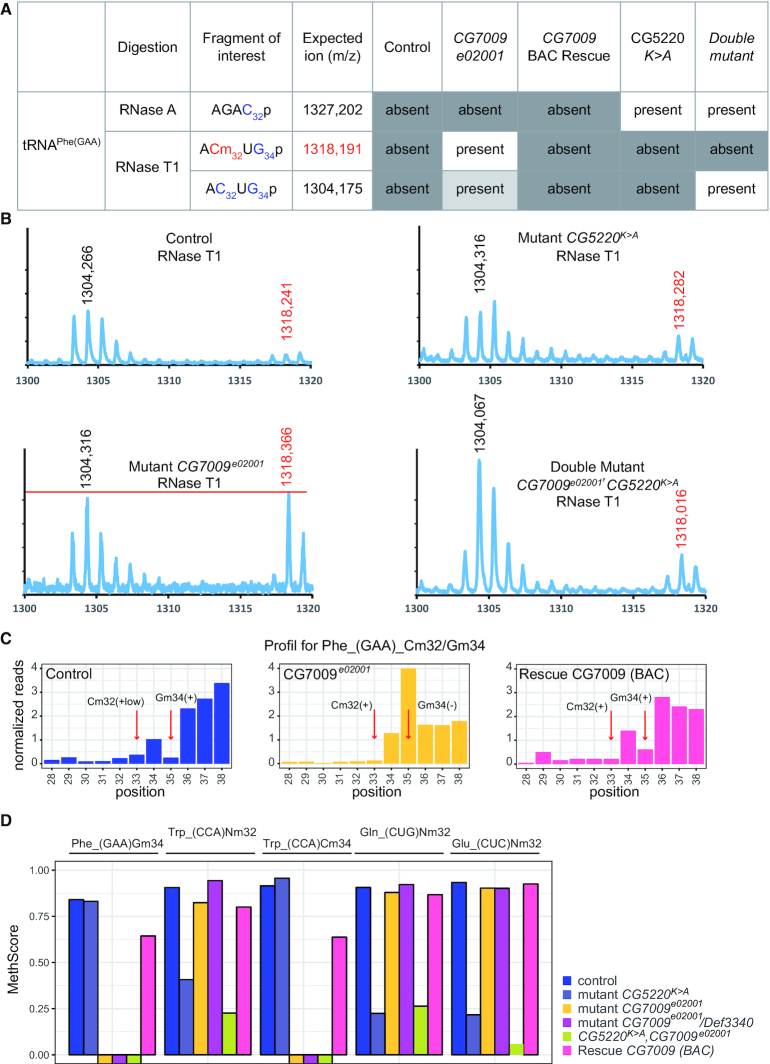
CG7009 & CG5220 are TRM7-like tRNA Nm MTases in *Drosophila*. (**A**) Presence (present) or absence (absent) of Nm-containing RNA fragments and their sizes (m/z in Daltons) upon RNase A or T1 digestion of tRNA^Phe(GAA)^ extracted from *Drosophila* adult heterozygous *CG7009^e02001^* mutants (Control), homozygous *CG7009^e02001^* mutants (CG7009 e02001), homozygous *CG7009* mutants rescue (CG7009 BAC Rescue), homozygous *CG5220^K>A^* mutants (CG5220 *K>A*), and double homozygous mutants *CG7009*^e02001^,*CG5220^K>A^* (Double mutant). (**B**) MALDI TOF-MS spectrum of fragments resulting from RNase T1 digestion of tRNA^Phe-(GAA)^ originating from heterozygous adult *CG7009^e02001^/+* mutants (Control), homozygous *CG7009^e02001^* mutants (Mutant *CG7009^e02001^*), red line indicates the maximum value (1318 Da) and is depicted for better comparison between the 2 peaks values. Homozygous *CG5220^K>A^* mutants (Mutant CG5220*^K>A^*) and double homozygous mutants *CG7009^e02001^*,*CG5220^K>A^* (Double Mutant *CG7009^e02001^*,*CG5220^K>A^*) as indicated. Relevant peaks are identified by their m/z values in Daltons (X-axis). (**C**) RiboMethSeq analysis of Nm at positions C_32_ and G_34_ in tRNA^Phe(GAA)^ from whole heterozygous adult *CG7009^e02001^/+* mutants (Control), homozygous *CG7009^e02001^* mutants (CG7009*^e02001^*) and rescued *CG7009^e02001^* mutants (Rescue CG7009 (BAC)) as indicated. Normalized cleavage efficiencies, calculated from combined 5′- and 3′-end coverage of tRNAs are shown for the neighboring nucleotides (+/– 5) of the respective ribose methylation position. The positions of interest (C_32_ and G_34_) in tRNA^Phe(GAA)^ are indicated by red arrows. Protection against cleavage is indicated (+): protected and as (–): not protected. Protection at Cm_32_ in control flies was only moderate, indicating incomplete tRNA methylation (+low). (**D**) Methylation scores (MethScore) for five 2′-*O*-methylated positions in tRNAs showing altered methylation in *CG5220* and/or *CG7009* indicated mutants. MethScore (Score C), representing the level of ribose methylation was calculated from protection profiles. Data are shown for positions 32 and 34 in different *D. melanogaster* tRNAs as measured in heterozygous adult *CG7009^e02001^/+* mutants (control), homozygous *CG5220^K>A^* mutant (mutant *CG5220^K>A^*), two independent genetic background mutants for *CG7009* (homozygous *CG7009^e02001^* or *trans*-heterozygous *CG7009^e02001^*/*Def3340* mutant), double homozygous *CG7009^e02001^*,*CG5220^K>A^* mutant and rescue BAC *CG7009^e02001^*/*Def3340* flies (Rescue CG7009 (BAC)). Score at Cm_32_ for tRNA^Phe(GAA)^ in control flies is only moderate (not shown and Figure 5C), indicating incomplete tRNA methylation.

To test whether CG7009 is an Nm-MTase, recombinant proteins were expressed and purified from *E. coli*. *In vitro* methylation assays using *in vitro*-synthesized *Drosophila* tRNA^Phe(GAA)^ did not reveal activity of recombinant CG7009 protein. In order to ascertain the predicted catalytic activities of CG7009 and CG5220, we analyzed the Nm methylation status of *Drosophila* tRNA^Phe^, which is a substrate of TRM7 in yeast and of FTSJ1 in human, using control, *CG7009^e02001^* and *CG5220^K>A^*mutant flies. We performed sequence-specific purification of tRNA^Phe^ using biotinylated DNA oligonucleotides coupled to streptavidin matrices followed by RNase digestion and MALDI-TOF mass spectrometry. RNase A has a preference for hydrolysis at pyrimidine residues, while RNase T1 is strictly guanosine-specific. Because Nm at a given nucleotide position (*n*) protects the adjacent 3′-phosphodiester bond to the neighboring nucleotide (position *n+1*) against nuclease attacks, various specific digestion products of *Drosophila* tRNA^Phe^ can be expected as a result of RNase A or RNase T1 activities. In addition, according to the reported modification profile of *Drosophila* tRNA^Phe^ (1,90) which includes Nm at C_32_ and G_34_, specific RNA fragments could thus be predicted (Figure 5A and Supplementary Figures S4A and B).

First, we determined RNA fragments that were obtained after RNase A hydrolysis of tRNA^Phe^, which should provide information on the Nm-modification status at C_32_. MALDI-TOF analysis revealed almost no RNA fragment of 1327.2 Da (AGAC_32_p fragment) in control flies indicating that C_32_ was modified with Nm thereby blocking RNase A activity at this position in tRNA^Phe^ from control and rescue flies (Figure 5A and Supplementary Figures S5A and B). This fragment increased significantly in *CG5220^K>A^* mutants suggesting loss of protection from RNase A activity in animals lacking CG5220. Interestingly, the increase in RNase A-mediated tRNA^Phe^ fragmentation observed in *CG5220^K>A^* mutants could only be moderately observed when using tRNA^Phe^ from *CG7009^e02001^,CG5220^K>A^* double mutant flies (Figure 5A and Supplementary Figure S5A) indicating that C_32_ protection from RNase A was largely independent of CG7009. In support of this notion, the *CG7009^e02001^* mutation alone did not affect the RNAse A digestion profiles when compared to control (Figure 5A and Supplementary Figure S5A) or BAC rescue *CG7009^e02001^* flies (Supplementary Figure S5B). These results indicated that CG5220, but not CG7009, harbors an activity that protects tRNA^Phe^ at C_32_ against RNase A digest, therefore making CG5220 the main candidate for an Nm-MTase at this position in *Drosophila*.

Next, we obtained RNase T1 digestion profiles to deduce the G_34_ modification status of tRNA^Phe^ in both control and Nm-MTase mutant flies. MALDI-TOF analysis showed a ACm_32_UG_34_p fragment (1318,1 Da) that could not be detected in control flies indicating that G_34_ was modified with Nm thereby blocking RNase T1 activity at this position in wild type tRNA^Phe^ (Figures 5A and B). This fragment increased significantly in *CG7009^e02001^* mutants suggesting loss of protection from RNase T1 activity in animals without CG7009. The RNase T1 digestion profiles from controls and *CG5220^K>A^* mutant flies were comparable (Figures 5A and B and Supplementary Figure S5B) indicating that CG7009 but not CG5220 is implicated in protecting G_34_ from RNase T1 digestion in tRNA^Phe^. Finally, digest of tRNA^Phe^ from *CG7009^e02001^*^,^*CG5220^K>A^* double mutant flies with RNase T1 produced a fragment (AC_32_UG_34_p) that was completely unmodified (1304 Da) suggesting that CG5220 and CG7009 are the responsible Nm-MTase activities that modify C_32_ and G_34_ in tRNA^Phe^, respectively (Figures 5A and B).

Collectively, these data demonstrated that genetic mutation of two candidate Nm-MTases in *Drosophila* resulted in the reciprocal loss of two conserved ACL modifications in tRNA^Phe^ strongly suggesting that CG5220 and CG7009 are indeed functional methyltransferases responsible for the deposition of Nm at C_32_ and G_34_ of tRNA^Phe^, respectively. Interestingly, our results also suggest that *Drosophila melanogaster*, and likely other *Drosophila* species, evolved two distinct TRM7 family members to ribose-methylate the ACL on substrate tRNAs (Figure 1D).

### Methylation specificity of both MTases depends on nucleotide position

To obtain a comprehensive picture of the Nm-MTase specificity for CG7009 and CG5220 *in vivo*, we performed RiboMethSeq analysis on *Drosophila* tRNAs. RiboMethSeq allows RNA-wide Nm detection based on random RNA fragmentation by alkaline hydrolysis followed by library preparation and sequencing (64,65). The presence or absence of Nm can be appreciated from characteristic coverage profiles of the 5′-/3′-ends of cDNAs. Since Nm residues protect the adjacent 3′-phosphodiester bond to the neighbouring nucleotide from hydrolysis, a gap in the coverage at the *n+1* position indicates the presence of a 2′-*O*-methylated nucleotide at position *n*. When analyzing the 2′-*O*-methylation status at position 34 for tRNA^Phe^ in control individuals, reads at position 35 (equals *n+1)* were under-represented in regard to their direct nucleotide neighbors (Figure 5C and Supplementary Figures S6A and B). This demonstrated that, Nm was present at G_34_ in *Drosophila* tRNA^Phe^ as previously reported (90) and as shown by MALDI-TOF MS analysis (Figure 5A). Similarly, RiboMethSeq profile analysis of *CG5220^K>A^* mutants indicated G_34_ to be methylated (Supplementary Figures S6A and B). Importantly, the presence of Nm at G_34_ in *CG5220^K>A^* mutant confirmed that CG5220 was not involved in the formation of ribose methylation at this position. On the contrary, in two different *CG7009* mutants, as well as in *CG7009^e02001^*, *CG5220^K>A^* double mutants, protection against hydrolysis at position 35 was totally abolished when compared to the control heterozygote profile (Figure 5C and Supplementary Figures S6A and B), confirming that CG7009 is the Nm-MTase for G_34_ of tRNA^Phe^ in *Drosophila* and that CG5220 alone is not able to methylate this position. Importantly, the expression of an additional gene copy of CG7009 in the *CG7009^e02001^* mutant background (Rescue CG7009 (BAC)) rescued the lost protection against hydrolysis at G_34_ of tRNA^Phe^ (Figure 5C and Supplementary Figure S6B). In addition, RiboMethSeq analysis was performed for position 33 of tRNA^Phe^ (*n* + 1 to the expected Nm at C_32_ (Figure 5A and (90)), which confirmed that CG5520, but not CG7009, was responsible for ribose methylation at position 32 on tRNA^Phe^ (Figure 5C and Supplementary Figures S6A and B).

Furthermore, RiboMethSeq analysis also identified other tRNAs potentially methylated by CG7009 and CG5220, some of which were already known as substrates of TRM7 orthologs in other species. For instance, we found CG7009-dependent methylation at position C_34_ and CG5520-dependent Nm at position C_32_ of tRNA^Trp^ (Figure 5D and Supplementary Figure S7). Strikingly, the methylated nucleotide at position 34 in tRNA^Trp^ of *Drosophila* is a cytosine, like in humans and in yeast (1,45,48,49). Importantly, RiboMethSeq scores clearly showed that CG7009 (and not CG5220) methylated this position (Figure 5D and Supplementary Figure S7) indicating that CG7009 can deposit Nm on G and C nucleotides. The same observation was made for CG7009-mediated methylation of C_34_ in tRNA^Leu(CAA)^, which was in agreement with previous data showing that FTSJ1 was responsible for depositing Nm at f5C_34_/hm5C_34_ in human tRNA^Leu(CAA)^ ((44) and Supplementary Figures S6C and S7). In addition, we identified previously unknown Nm-MTase substrate tRNAs. For instance, RiboMethSeq uncovered CG5220-dependent methylation of tRNA^Gln^ and tRNA^Glu^ at position C_32_ (Figure 5D and Supplementary Figure S7). 2′-*O*-methylated C_32_ in tRNA^Glu(UUC)^ had previously been reported in *Drosophila* (1,91). Interestingly, cytosine 32 was also reported to be 2′-*O*-methylated in human tRNA^Gln^ by a yet unidentified enzyme (1). Our data thus suggest that the human ortholog of CG5220, FTSJ1, may be the Nm-MTase responsible for the modification at this position.

Altogether, detailed RiboMethSeq analysis confirmed the MALDI-TOF MS results (Figure 5A, B and Supplementary Figure S5), demonstrating that CG5220 is specialized for depositing Nm at C_32_ nucleotides while CG7009 is responsible for modifying the wobble position. Furthermore, the discovery of additional tRNA substrates (Figure 5D and Supplementary Figure S7) for both Nm-MTases suggested that their respective specificity is dependent on the position rather than on the nature of nucleotide (C, U or G).

### CG33172 is part of the Nm–MTase complex

**Figure 6.**
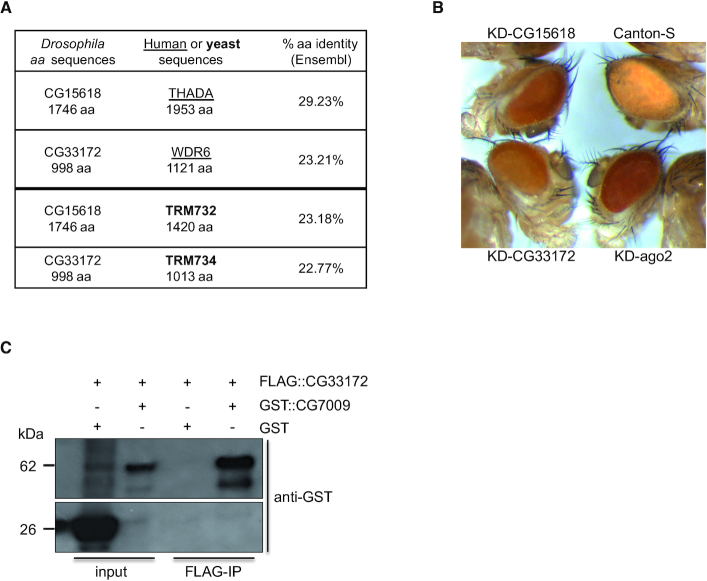
CG33172 is a partner of the Nm-MTase complex in *Drosophila*. (**A**) Percentage of amino acid (aa) identity between CG15618, human THADA and yeast TRM732, and between CG33172, human WDR6 and yeast TRM734 (RTT10). Alignment was performed using BLAST/ BLAT tool at www.ensembl.org. (**B**) CG33172 and CG15618 modulate Ago2-dependent silencing in adults flies. *CG33172* and *CG15618* expression was knocked down by using UAS-RNAi lines and eye- specific *GMR*-Gal4, [w-] driver (indicated as KD), as in Figure 2B. Canton-S (wild type, [w+]) and *Ago2* KD were used as controls. A darker eye coloration than Canton-expressing *automiW* lines (top right) indicates that the miRNAs of the sensor are failing to inactivate the *white* gene through impaired miRNA biogenesis or/and Ago2-dependent silencing. (**C**)CG33172 interacts with CG7009 *in vitro*. Co-immunoprecipitation of recombinant and epitope-tagged CG7009 and CG33172 after co-expression in bacteria. Western blotting using anti-GST antibody on protein extracts from input fractions co-expressing GST::CG7009 and FLAG::CG33172 and after FLAG-IP; Lower panel, Anti-GST WB reveals a GST ‘alone’ signal in the co-expressed GST and FLAG::CG33172. Inputs correspond to 10% of 10 μg of protein eluates. The expected protein sizes are 26 kDa (GST) and 62 kDa (GST::CG7009). WB, western blot; kDa, kilodaltons.

Yeast TRM7 associates with two distinct proteins that are required for its catalytic activity (45,46). Deposition of Nm at C_32_ by TRM7 is supported by binding to TRM732 while the interaction with TRM734 is necessary for addition of Nm at position 34. THADA and WDR6 are the orthologs of TRM732 and TRM734 in humans, respectively, and their interactions with FTSJ1 are conserved (49). In *Drosophila*, CG15618, also known as DmTHADA (92), is the potential ortholog of TRM732 and THADA, while CG33172 is the putative ortholog of TRM734 and WDR6 (Figure 6A). Importantly, CG33172, TRM734 and WDR6 are members of the WD40-repeat-containing domain superfamily that contains also the human protein WDR4, another tRNA-MTase cofactor that, like FTSJ1, when mutated is associated with neurodevelopmental disorders (93,94).

The use of the *automiW* sensor combined with dsRNA-mediated knockdown of CG15618 and CG33172 in the *Drosophila* eye recapitulated the Ago2-mediated small RNA silencing failure observed in *CG7009* and *CG5220* mutants (Figure 6B). Interestingly, dsRNA-mediated knockdown of CG33172 using the *Gypsy-LacZ* sensor also recapitulated the somatic piRNA-mediated silencing failure observed in both *CG7009* and *CG5220* mutants (Figure 2F), indicating genetic interactions between CG7009/ CG5220-mediated functions and these gene products.

In order to test for physical interactions between CG7009, CG15618 and CG33172, we cloned FLAG-tagged CG15618 and CG33172 with the aim of co-expressing these proteins along with GST::CG7009 in bacteria. While co-expression of FLAG::CG15618 was technically challenging due to the size of this protein (197 kDa), FLAG::CG33172 could be expressed and immunoprecipitated using anti-FLAG antibodies. The precipitate was tested for the presence of GST::CG7009 by using western blotting and anti-GST antibodies. The results showed that FLAG::CG33172 co-precipitated with GST-CG7009 but not GST alone indicating a direct interaction between these two proteins (Figure 6C). Collectively, these observations suggested the existence of an Nm-MTase complex containing CG7009 and at least one accessory protein, CG33172, which might be required for depositing Nm at position 34 on selected tRNAs.

### Nm limits endonucleolytic cleavage of tRNA^Phe^ and stabilizes tRNA^Phe^ fragments

**Figure 7.**
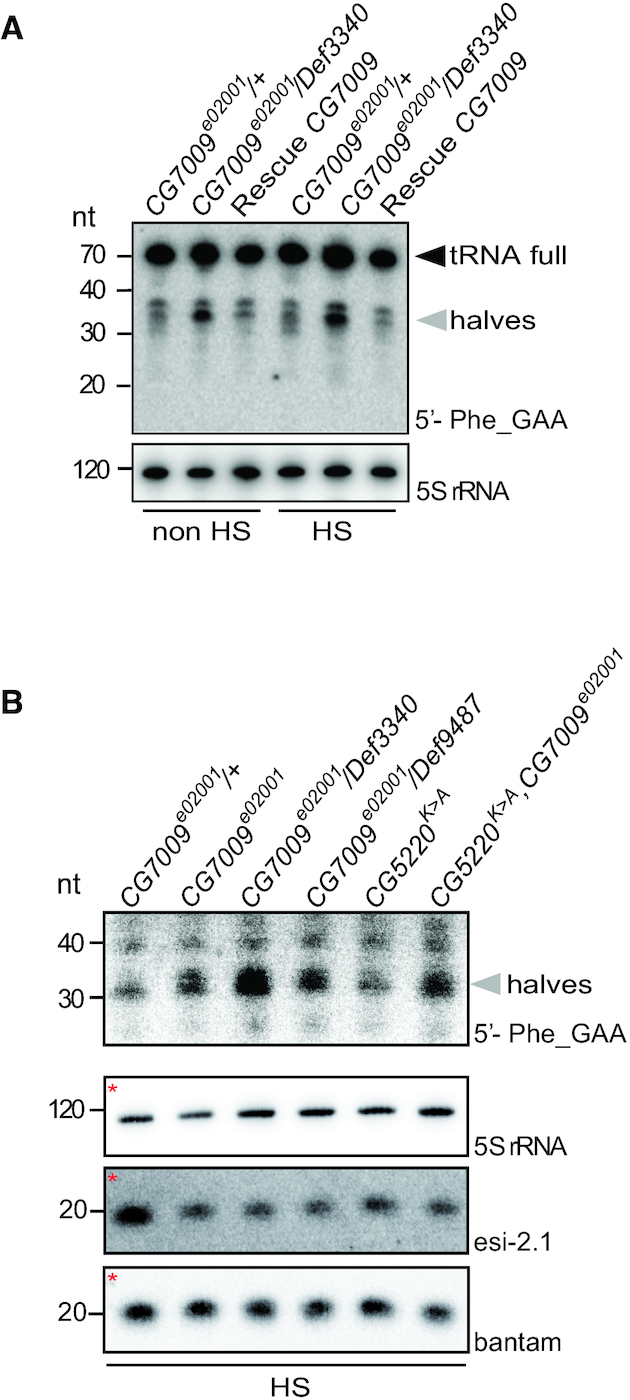
Nm limits endonucleolytic cleavage of tRNA^Phe^. (**A**) Northern blot characterization of 5′- tRNA^Phe(GAA)^-derived tRFs. Northern blot on total RNAs extracted from whole heterozygous *CG7009^e02001^* control flies (*CG7009^e02001^*/+), *trans*-heterozygous for *CG7009^e02001^*/*Def3340* or rescued mutants for *CG7009* (rescue *CG7009*) as indicated using a 5′-tRNA^Phe(GAA)^ -specific probe and a 5S rRNA probe as loading and transfer control. Mature tRNA^Phe^ size is 73 nt (full length). 5′-tRNA^Phe^-derived tRNA fragments (5′-tRF^Phe^) were detected at ∼35 nt (halves). The same experiment was performed on heat-shocked flies (one hour at 37°C in a water bath), RNAs were extracted after 5 h at 25°C (indicated as HS, heat shock). nt: nucleotide. (**B**) The same experiment as in Figure 7A above was performed on heat-shocked flies (one hour at 37°C in a water bath). The same membrane as shown in Figure 2D was stripped and reprobed with a tRNA^Phe(GAA)^ -specific probe. Figures 7B and 2D thus contain identical images (marked with *) for bantam, 5S and esi-2.1 for a better comparison. RNAs were extracted after 5 hours of recovery at 25°C (indicated as HS, heat shock) with indicated genotypes. Double homozygous mutant is indicated as *CG7009^e02001^*,*CG5220^K>A^*. Homozygous mutant for *CG7009* is indicated as *CG7009^e02001^*. Homozygous mutant for *CG5220* is indicated as *CG5220^K>A^*. *Trans*-heterozygous mutants for *CG7009* alleles are indicated as *CG7009^e02001^*/*Def3340* and *CG7009^e02001^*/*Def9487* nt: nucleotide.

We next addressed the mechanisms underlying the defects in the Ago2-mediated small RNA silencing activity observed in Nm-MTase mutant flies. It has been reported that loss of m^5^C and Queuosine from specific tRNAs resulted in increased tRNA fragmentation in *Drosophila (**95**)* and mammals (96,97). Furthermore, it was proposed that tRNA fragments (tRFs) could affect small RNA silencing pathways through binding to Dicer and Argonaute proteins thereby reducing their activity (95,98–101). In addition, during the preparation of this manuscript, a study showed that Nm_34_ protected tRNA^Met(CAT)^ from endonucleolytic cleavage by stress-induced angiogenin in human cells (18). We therefore tested if lack of Nm at positions 32 and 34 of tRNA^Phe^ affected its endonucleolytic cleavage during heat stress conditions. A heterozygous *CG7009^e02001^* mutant (control), a CG7009 trans-heterozygous mutant (*CG7009^e02001^*/*Def3340*) and the rescue line for CG7009 (Rescue *CG7009*) were analyzed by northern blotting with a specific probe complementary to the 5′- end of tRNA^Phe^ before and after heat shock exposure. Two clear hybridization signals were observed, corresponding to mature tRNAs (∼70 nt) and tRFs (∼35 nt, Figure 7A). tRNA fragmentation increased significantly in *CG7009^e02001^*/*Def3340* mutants. Importantly, increased tRNA fragmentation was rescued in Rescue *CG7009* flies (Figure 7A), demonstrating that CG7009 function affected tRNA fragmentation of tRNA^Phe^. Of note, global steady state levels of mature tRNAs were not affected in *CG7009^e02001^*/*Def3340* mutants (Figure 7A), suggesting limited pan-translational defects in flies without functional CG7009, while not excluding defective translation of specific proteins. Interestingly, we did not observe heat stress-dependent effects on tRNA fragmentation in *CG7009^e02001^*/*Def3340* nor in other *CG7009* mutant combinations (Figures 7A and B), indicating that increased tRNA fragmentation in *CG7009* mutants might be the result of increased tRNA^Phe^ turnover. Furthermore, when compared to *CG7009* single mutants, we did not observe increased tRNA^Phe^ fragmentation in *CG5220^K>A^* single nor in *CG7009/CG5220^K>A^* double mutants (Figure 7B), suggesting that Nm at position G_34_, and not C_32_, limits fragmentation of tRNA^Phe^, while 3′ terminal Cm_32_ might exert a stabilizing effect on tRFs (tRF^Phe^Cm_32_) that were produced in *CG7009* mutants.

## DISCUSSION

While performing an RNAi genome-wide screen for modulators of miRNA biogenesis or Ago2-dependent small RNA silencing in *Drosophila*, we identified a previously uncharacterized gene (*CG7009*) with sequence homology to Nm-MTases of the TRM7/FTSJ1 subfamily of MTase proteins. Surprisingly, through subsequent sequence analysis, we also identified CG5220 in *Drosophila*, which although sharing considerable sequence homology with CG7009 was not uncovered by the genetic screen in S2 cells. Furthermore, when re-testing (both visually and by western blotting) the *automiG* read-out (GFP expression) upon efficient CG5220 knockdown in S2 cells, we obtained variable and inconclusive results, which supported the fact that CG5220 was not uncovered as modulator of miRNA biogenesis or Ago2-dependent small RNA silencing by the original screen in S2 cells. However, since RNAi-mediated knockdown of CG5220 expression and a genetic mutation of the predicted catalytic motif in CG5220 (CG5220^K>A^ mutant) affected both the *automiW* sensor in adult *Drosophila* eyes and also sensors reporting on piRNA-mediated TE silencing in the germline, we believe that CG5220 function might be required only in certain tissues but not in embryo-derived S2 cells.

By characterizing the molecular function of these predicted Nm-MTases, we demonstrated that both genes encode RNA methyltransferases depositing Nm on tRNAs while displaying specialized activity at two distinct positions in the ACL. These findings reveal that, in contrast to yeast and humans, which encode only one Nm-MTase gene capable of methylating the ACL, *D. melanogaster* has evolved two distinct enzymes, each specialized in the methylation of only one position in the ACL of conserved tRNA targets. Interestingly, it appears also that other *Drosophila* species evolved and maintained these closely related TRM7/FTSJ1 paralogs. Mass spectrometry analysis and RiboMethSeq confirmed this peculiarity, raising the possibility that independent Nm deposition in the ACL of specific tRNAs by two enzymes, rather than one, might be functionally significant, in particular since expression and activity of both enzymes can be independently regulated. Importantly, our analysis also reports novel substrates of the TRM7 subfamily of Nm MTases.

Interestingly, and in agreement with studies on various RNA modification enzymes in other organisms, Nm-MTases in *Drosophila* are not required for organismal viability or fertility. However, Nm-MTases mutants displayed reduced lifespan and behavioral phenotypes manifested as general mobility defects (Figure 4). Although the use of the genetic double mutants *CG5220^K>A^*, *CG7009^e02001^* confirmed the outcome of the double KD experiments, it remains to be seen whether the effects on lifespan can be solely attributed to the loss of the catalytic function of CG5220 and not CG7009, because the genetic background of the presented experiments (Figure 4B) differed slightly in terms of generations times after isogenization.

Abolishing the catalytic function of both genes in the same animal did not reveal additional morphological mutant phenotypes with the notable exception of a reduction in size and weight, highlighting a potential role of these Nm-MTases in specific, but not general, translational control as previously reported for *trm7* mutant yeast (46,48,51,52) and as recently highlighted for internally deposited Nm on specific mRNA in humans (27).

Nm modifications in the ACL of specific tRNAs can affect translational efficiency and fidelity (51,52,102). Consistently, Nm deposition in mRNA also affects translation through interference with tRNA decoding efficiency and thus can potentially rewire the genetic code (27,103,104). Interestingly, it was recently proposed that TRM7 can also methylate substrates that are not tRNAs including mRNAs in yeast (25) suggesting that TRM7 family members can act as multi-substrate Nm-MTases, thereby modulating translation through modification of codons (mRNA) and anticodons (tRNA). Importantly, loss of Nm at tRNA positions 32 and 34 in *trm7* mutant yeast activated the general amino acid control pathway (GAAC, (52)), affected translation rates and, consequently, cell growth (46,48). Thus, the observed reduction of size and mass (Figure 2A) in flies without TRM7 family members supports the hypothesis that *CG5220* and *CG7009* mutations affect translational efficiency in *Drosophila*.

Importantly, a lack of Nm at the wobble position in *CG7009* mutants affected tRNA fragmentation patterns. tRNA fragmentation is a conserved response to various stress conditions affecting protein synthesis, apoptosis signalling and the modulation of small non-coding RNA pathways (105–108). An influence of internal Nm modifications on tRNA stability has only been described very recently (18). In support of the notion that Nm in tRNAs might modulate their stability, we found that Nm_34_ is protective against tRNA fragmentation in the ACL. However, in contrast to the increase in detectable tRFs in *CG7009* mutants, the lower level of tRFs in *CG7009*, *CG5220* double mutants suggests that CG5220 function affects tRNA fragment abundance positively once produced. Furthermore, since *CG5220* mutants did not display tRFs, we propose that tRFs produced by ACL cleavage in *CG7009* mutants may be stabilized through the existence of a 3′ terminal Nm at position 32 (likely deposited by CG5220). Since Hen1-mediated deposition of Nm at RNA 3′- termini (22,109) stabilizes small RNAs in various organisms, such a mechanism could explain the abundance and apparent stability of tRFs in *CG7009* single mutants, in contrast to the low abundance of tRFs in *CG5220* single and *CG7009*, *CG5220* double mutants.

Importantly, loss of function of either CG7009 or both Nm-MTases impaired Ago2- as well as Piwi-dependent small RNA silencing pathways *in vivo*. Furthermore, *DCV* infection assays and lower esi-2.1 production in adult Nm-MTase mutant flies confirmed a function for CG7009 and CG5220 as regulators of siRNA pathway-mediated mobile element control. In addition, the observation that the total miRNA population was reduced by 10% in *CG7009* mutants when compared to control (Figure 3A), indicated that Nm-MTases function affected small RNA silencing pathways in a pleiotropic fashion and suggested that these Nm-MTases genes could even act upstream of small RNA biogenesis and function. Indeed, both RNA and small RNA sequencing analysis suggested that the manifestation of these phenotypes could partially be due to the transcriptional downregulation of *Ago2* mRNA in *CG7009* mutant flies or after knockdown of CG7009 in S2 cells. On the other hand, tRFs can associate with Dicer, Argonaute and Piwi proteins (98–101,110). One potential consequence of such interactions could be a reduction in the capacity of small RNA pathway components to process or bind to canonical RNA substrates. Indeed, tRF-mediated titration of proteins away from canonical substrates has been reported (95,111–113).

Finally, our study identifies CG33172 as a binding partner of CG7009. Interestingly, the yeast ortholog of CG33172, TRM734, was reported to control the steady state levels of TE as does TRM7 (114). Here, we have shown that the *Drosophila* orthologs of TRM734 and TRM7 (CG331772 and CG7009, CG5220, respectively) also affected TEs through siRNA and piRNA-mediated silencing pathways. Furthermore, sncRNA pathways and TE expression (R2 and especially *gyspy)* in *Drosophila* glial and neuronal cells have already been associated with decreased lifespan as well as with the manifestation of neurodegenerative disease (115–117).

Importantly, mutations in human WDR4 impaired tRNA m^7^G_46_ methylation and caused microcephalic primordial dwarfism (94). CG33172 belongs to the WD-Repeat (WDR) family of proteins and is the ortholog of human WDR6. Interestingly, both WDR6 and FTSJ1 were identified as principal human host restriction factors against *vaccinia* virus indicating that this Nm-MTase complex functions at the interface of host-virus interactions (118). In support of the notion that Nm modifications modulate mobile and repeat element control, human Nm-MTase FTSJ3 can be hijacked by HIV-1 to methylate viral mRNAs resulting in avoidance of being sensed by the host innate immune system (26).

Our study thus strongly supports the emerging notion that an important biological impact of Nm-MTase activity is mobile element control affecting TEs and viruses. Importantly, our results in *Drosophila* also indicate that the molecular machinery necessary for depositing Nm in tRNAs and the associated physiological importance are conserved throughout evolution.

In summary, this study provides a comprehensive *in vivo* characterization of two Nm-MTases and associated functions in *D. melanogaster*, demonstrating the importance of enzymes of the TRM7/FTSJ1 family in contributing to small non-coding RNA silencing pathways. The peculiarity of *Drosophila* species having evolved two TRM7/FTSJ1 gene products with specialized activity towards specific positions in tRNAs for *D. melanogaster* strongly suggests that Nm in other *Drosophila* species is deposited at ACLs by two TRM7/FTSJ1 enzymes. Importantly, our results also support the notion that the stability of particular tRFs could depend on their RNA modification status. Regarding the respective specificity of ACL methylation in the *Drosophila* clade, we propose to rename the identified *Drosophila* genes as *dTrm7_34* (for *CG7009*) and *dTrm7_32* (for *CG5220*).

## DATA AVAILABILITY

The RNA sequencing data discussed in this publication have been deposited in NCBI’s Gene Expression Omnibus (119) and are accessible through GEO Series accession number GSE134354 (https://www.ncbi.nlm.nih.gov/geo/query/acc.cgi?acc=GSE134354). The small RNA sequencing data discussed in this publication have been deposited in the European Nucleotide Archive (ENA) at EMBL-EBI under accession number PRJEB35301 (https://www.ebi.ac.uk/ena/data/view/PRJEB35301).

## Supplementary Material

gkaa002_Supplemental_Files
