# Supplementary material for: tRNA 2′-O-methylation by a duo of TRM7/FTSJ1 proteins modulates small RNA silencing in Drosophila
Source: Nucleic Acids Res. 2020 Jan 16;48(4):2050–72. doi: 10.1093/nar/gkaa002 (PMC7038984; doi:10.1093/nar/gkaa002)
Supplement: gkaa002_Supplemental_Files [file gkaa002_supplemental_files.zip › Sup_Figures and Legends S1_S7.pdf]

A

#	Function (known or predicted)	Gene name	CG number
1	protein histidine phosphatase activity	*janA*	CG7933
2	Unknown		CG12688
3	Hsp90 protein binding; chaperone binding; prostaglandin-E synthase activity.	*p23*	CG16817
4	rRNA processing; translation.		CG1789
5	Unknown		CG18537
6	Unknown		CG42240
7	Unknown		CG5866
8	mRNA splicing/ binding		CG3542
9	protein transporter	*AP-50*	CG7057
10	phospholipase A1		CG6283
11	Electron transport	*wal*	CG8996
12	alpha-amylase activity	*Amy-p/Amy-d*	CG18730
13	Antibacterial peptide metabolism	*Anp*	CG1361
14	tRNA/ rRNA methyltransferase		CG7009
15	Sensorial perception odorant binding	*Obp19c*	CG15457
16	response to virus; post-mating behavior	*EPebIII*	CG11390
17	Calcium ion binding		CG5890
18	RNA silencing	*Drosha*	CG8730
19	RNA silencing	*pasha*	CG1800
20	RNA silencing	*Ago2*	CG7439
21	RNA silencing	*Droj2*	CG8863

C

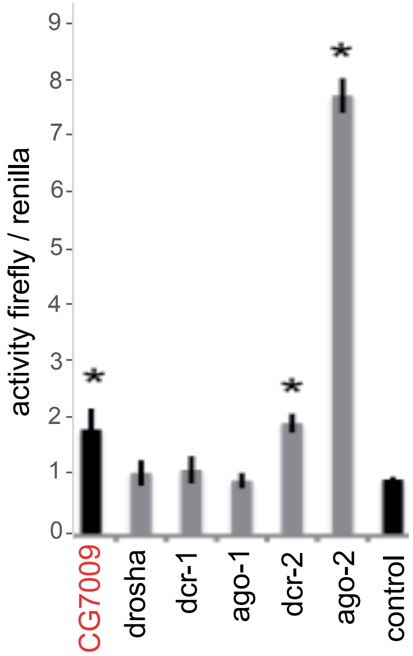

D

	Log2 fold changes
ND4	-1
Cyt-b	-0,91
ND1	-0,83
CoI	-0,82
CoII	-0,76
Ago2	-0,7
ND5	-0,66
ATPase6	-0,62
lrRNA	-0,52
exd	-0,34

B

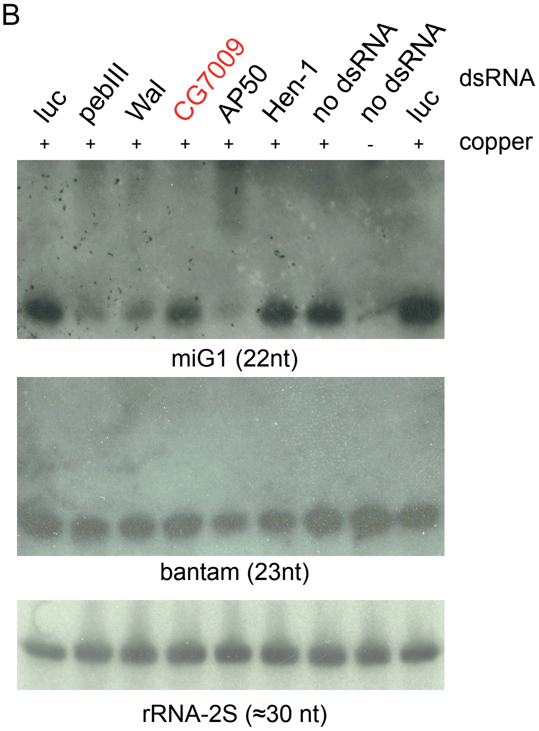

E

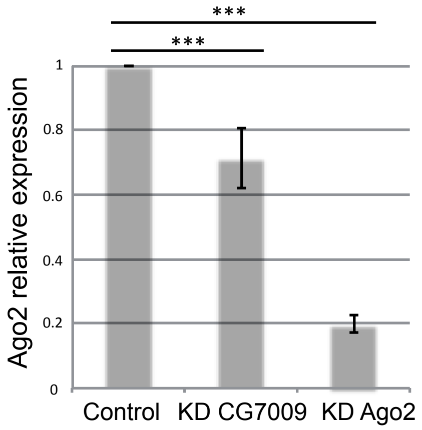

Sup_Figure.1

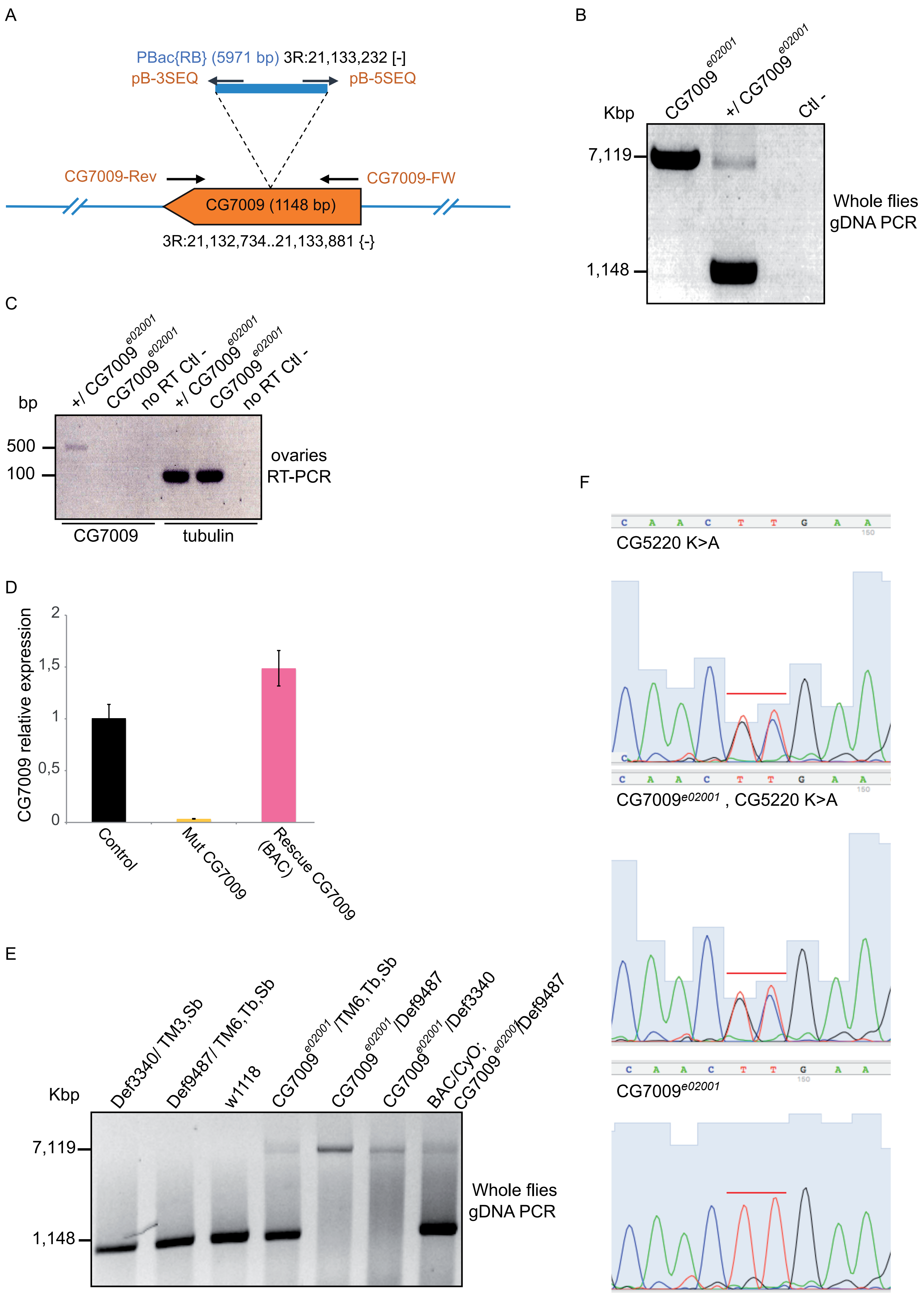
A
PBac{RB} (5971 bp) 3R:21,133,232 [-]
pB-3SEQ
pB-5SEQ
CG7009-Rev
CG7009-FW
CG7009 (1148 bp)
3R:21,132,734..21,133,881 {-}
B
Kbp
CG7009 e02001
+/ CG7009 e02001
Ctl -
7,119
1,148
Whole flies gDNA PCR
C
bp
+/ CG7009 e02001
CG7009 e02001
no RT Ctl -
+/ CG7009 e02001
CG7009 e02001
no RT Ctl -
500
100
ovaries RT-PCR
CG7009
tubulin
D
CG7009 relative expression
2
1,5
1
0,5
0
Control
Mut CG7009
Rescue CG7009 (BAC)
E
Kbp
Def3340/ TM3,Sb
Def9487/ TM6,Tb,Sb
w1118
CG7009 e02001 /TM6,Tb,Sb
CG7009 e02001 /Def9487
CG7009 e02001 /Def3340
BAC/CyO; CG7009 e02001/Def9487
7,119
1,148
Whole flies gDNA PCR
F
C A A C T T G A A
CG5220 K>A
C A A C T T G A A
CG7009 e02001 , CG5220 K>A
C A A C T T G A A
CG7009 e02001

Sup_Figure.2

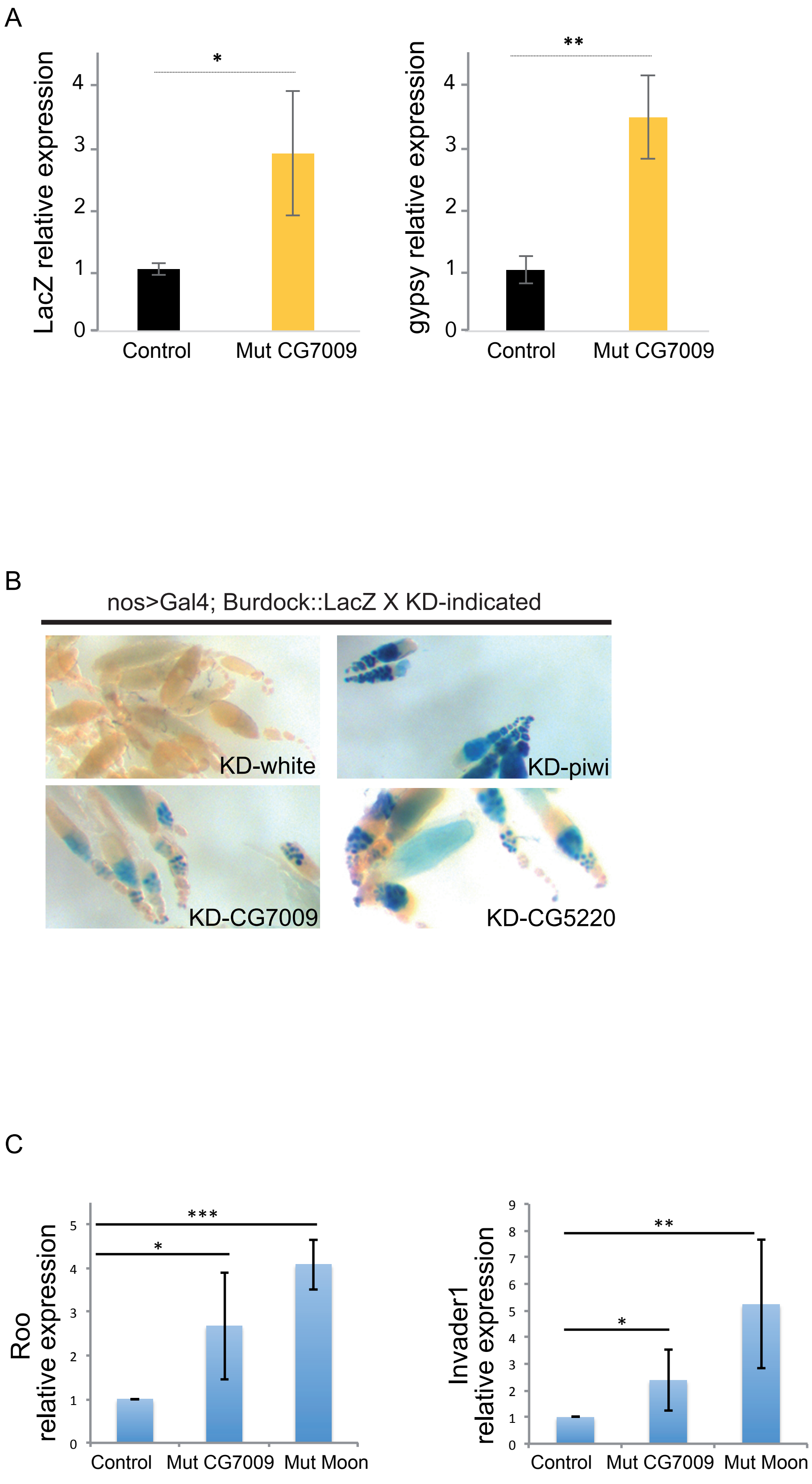
A
LacZ relative expression
gypsy relative expression
Control
Mut CG7009
B
nos>Gal4; Burdock::LacZ X KD-indicated
KD-white
KD-piwi
KD-CG7009
KD-CG5220
C
Roo relative expression
Invader1 relative expression
R2 relative expression
Control
Mut CG7009
Mut Moon

Sup_Figure.3

A

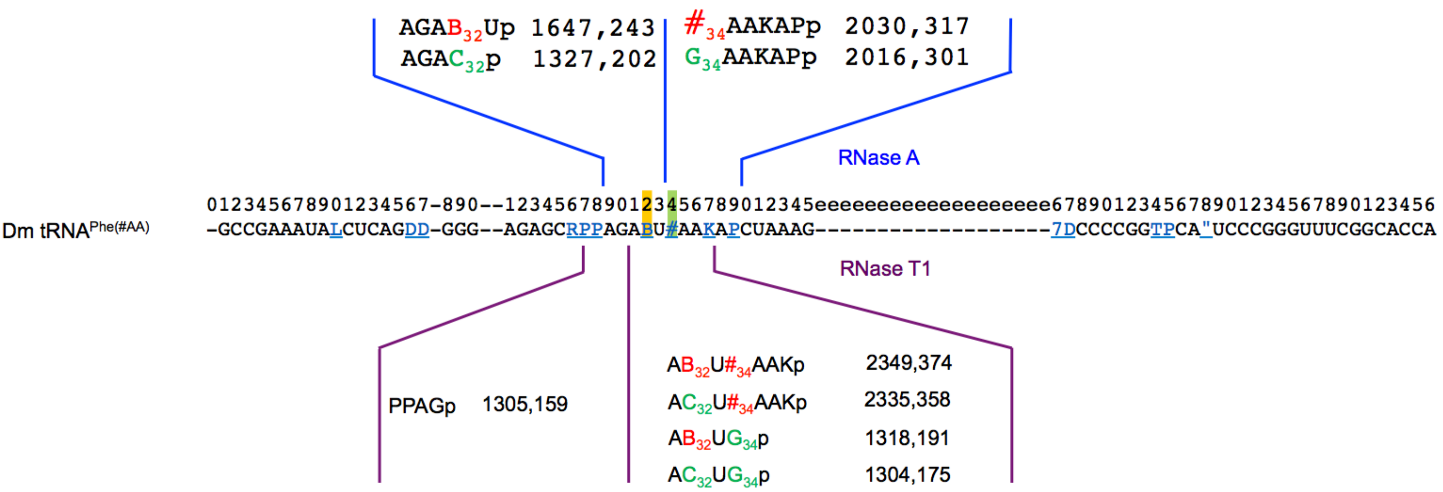

B

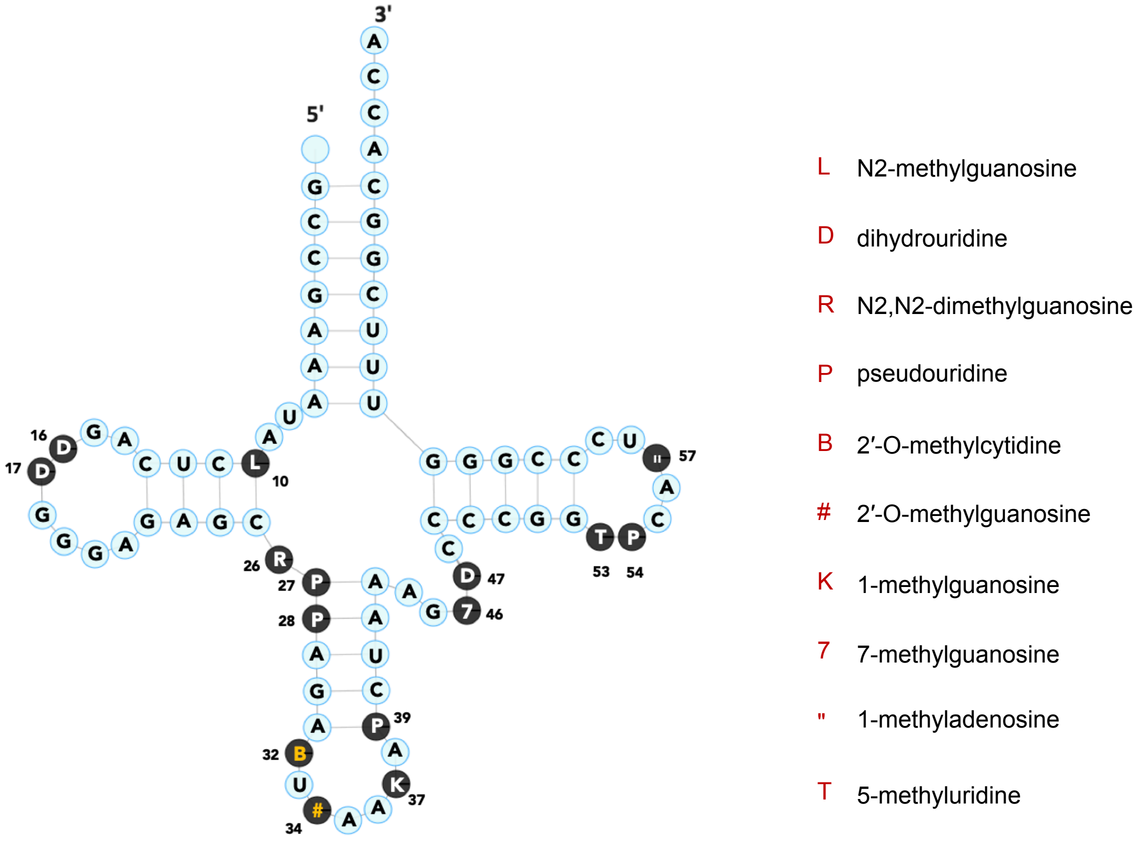

Sup_Figure 4

A

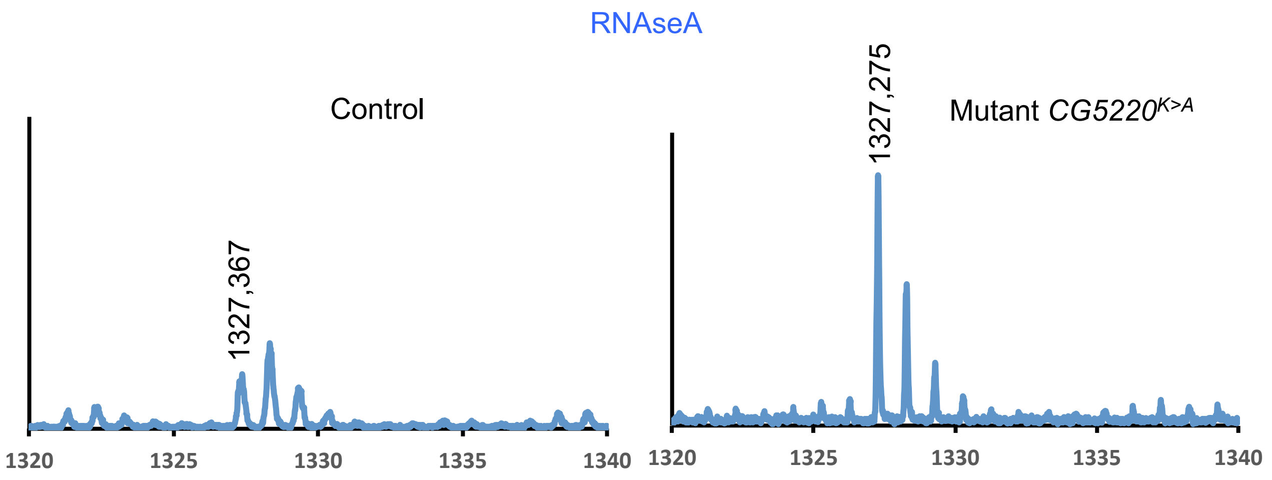

B

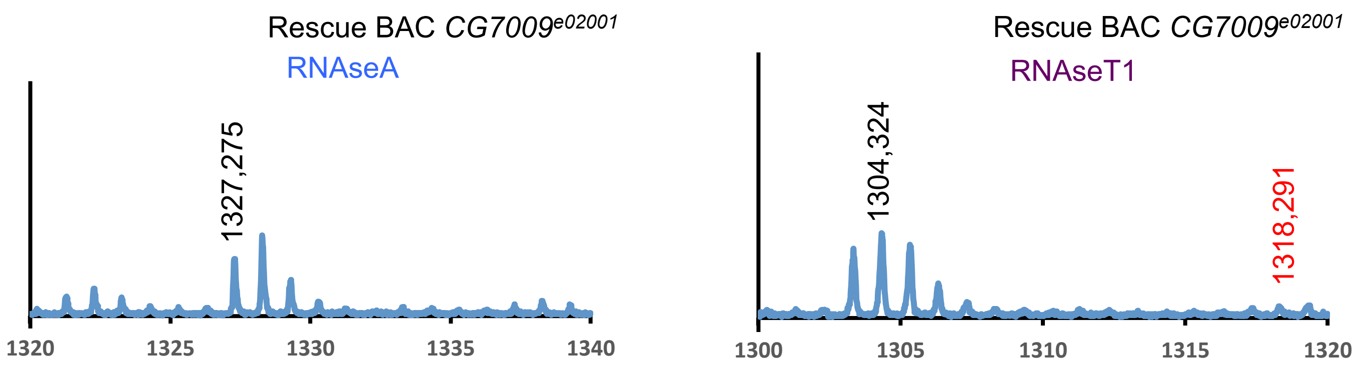

Sup_Figure 5

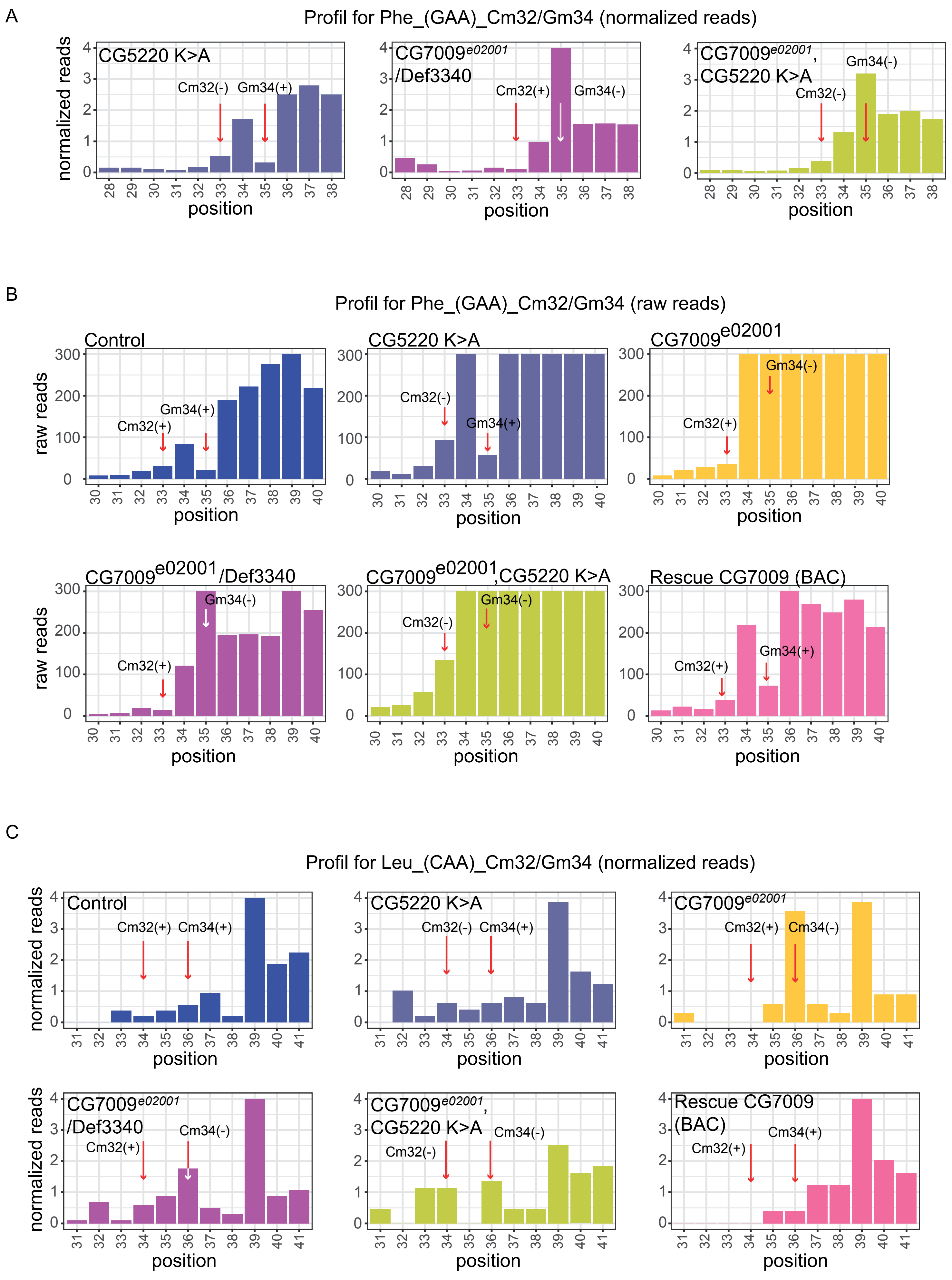
A
Profil for Phe_(GAA)_Cm32/Gm34 (normalized reads)
normalized reads
CG5220 K>A
Cm32(-) Gm34(+)
CG7009e02001 /Def3340
Cm32(+) Gm34(-)
CG7009e02001, CG5220 K>A
Cm32(-) Gm34(-)
position
B
Profil for Phe_(GAA)_Cm32/Gm34 (raw reads)
raw reads
Control
Cm32(+) Gm34(+)
CG5220 K>A
Cm32(-) Gm34(+)
CG7009e02001
Cm32(+) Gm34(-)
CG7009e02001/Def3340
Cm32(+) Gm34(-)
CG7009e02001,CG5220 K>A
Cm32(-) Gm34(-)
Rescue CG7009 (BAC)
Cm32(+) Gm34(+)
position
C
Profil for Leu_(CAA)_Cm32/Gm34 (normalized reads)
normalized reads
Control
Cm32(+) Cm34(+)
CG5220 K>A
Cm32(-) Cm34(+)
CG7009e02001
Cm32(+) Cm34(-)
CG7009e02001 /Def3340
Cm32(+) Cm34(-)
CG7009e02001, CG5220 K>A
Cm32(-) Cm34(-)
Rescue CG7009 (BAC)
Cm32(+) Cm34(+)
position

Sup_Figure.6

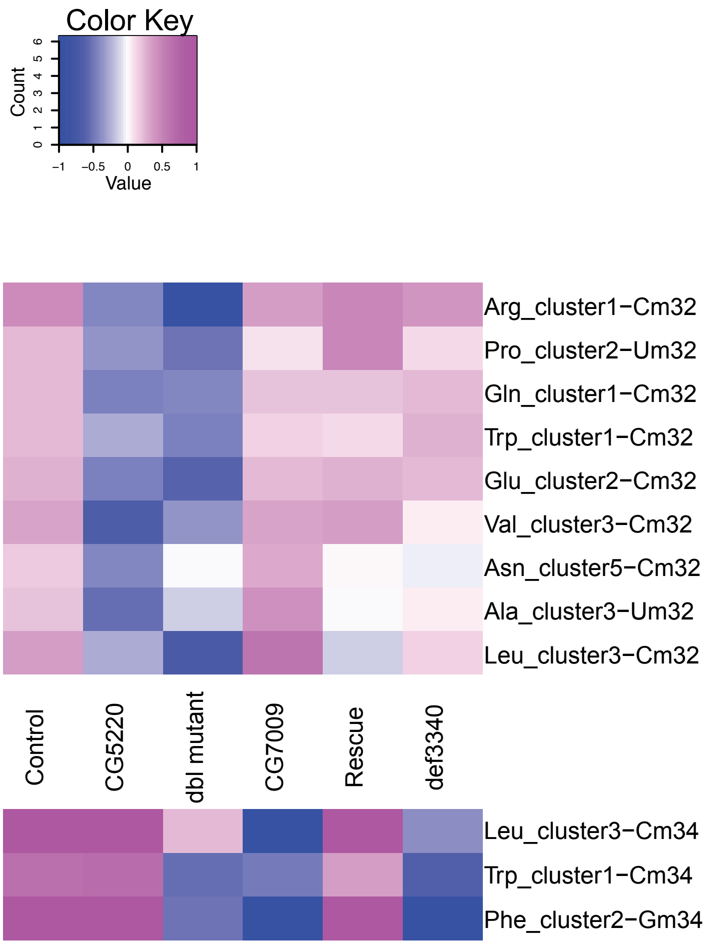
Color Key
Count
0 1 2 3 4 5 6
−1 −0.5 0 0.5 1
Value
Arg_cluster1−Cm32
Pro_cluster2−Um32
Gln_cluster1−Cm32
Trp_cluster1−Cm32
Glu_cluster2−Cm32
Val_cluster3−Cm32
Asn_cluster5−Cm32
Ala_cluster3−Um32
Leu_cluster3−Cm32
Control
CG5220
dbl mutant
CG7009
Rescue
def3340
Leu_cluster3−Cm34
Trp_cluster1−Cm34
Phe_cluster2−Gm34

Sup_Figure.7

Supplementary Figure Legend

Figure S1.

A, List of the validated genes in the RNAi genome-wide screen. Indicated are the genes that scored positively in the *automiG* screen, their described or predicted function in FlyBase, the gene name (when available), and the CG number, according to FlyBase. Blue colored names indicate genes already known in RNA silencing pathways. Red point to CG7009.

B, Northern blot characterization of miG1 Ago2-loaded miRNA in KD *CG7009 automiG* S2 cells. Northern blot on total RNAs extracted from *automiG* transfected S2 cells induced (+) or not (-) with copper sulfate for *automiG* expression and KD using dsRNA transfection on indicated genes were performed using miG1 (Ago2 loaded) or bantam (Ago1 loaded) miRNAs specific probes and a 2S rRNA probe as loading and transfer control. nt: nucleotide.

C, siRNA sensor (80, 81) reveal failure in Dcr2/ Ago2-dependent siRNA pathway. After 4 days of treatment with a dsRNA specifically inactivating the indicated gene, S2 cells were co-transfected with 2 vectors expressing firefly and Renilla (control) luciferases in addition to dsRNA targeting firefly luciferase (dsRNA Fluc). Firefly and renilla activities were measured 48 hours after transfection. The averages of the ratios of firefly/renilla activity for three independent experiments with standard deviations of the means.d are represented, the average for the dsRNA GFP (control) being set at 1. * indicates $p < 0.05$ *versus* control in a Student's t-test.

D, Ago2 mRNA is downregulated upon *CG7009* KD. The heat map shows the top 10 differentially expressed genes in S2 cells upon *CG7009* KD versus control (*LacZ* KD) in a RNA sequencing experiment. log2 Fold Change is indicated (padj = FDR-adjusted *p*-value for Ago2 is $7.73e^{-118}$). Red to yellow heat map colored table (Red: most down-regulated; yellow, less down-regulated). KD of *CG7009* led to the deregulation of 173 genes including 45 up and 128 down. 110 genes were deregulated with a corrected *p*-value (Benjamini and Hochberg, FDR) < 0.01.

E, Ago2 mRNA is downregulated upon *CG7009* KD. RT-qPCR using *Ago2*-specific primers on *automiG*-expressing S2 cells KD for *CG7009* (KD CG7009), *Ago2* (KD Ago2) and mock (Control). Error bars represent the standard deviation (s.d.) between four independent biological replicates. ****p-value* < 0.001 in a Student's t-test.

Figure S2.

A, schematic representation of the $CG7009^{e02001}$ allele showing the genomic location of the insertion and the size of the PBac{RB} transgenic transposon. The four hybridizing

oligonucleotides used in the genomic PCR analysis are represented with arrows representing their annealing locations and orientation (sequences in *Primers and Probes section*).

B, Agarose gel electrophoretic separation of the PCR reaction made on gDNA (genomic DNA) of adult flies *CG7009* homozygous or heterozygous for the mutant allele *CG7009*^{e02001} or Ctl - (no DNA) using CG7009-FW and CG7009-Rev primers. Expected sizes in base paired (Kbp) are indicated on the left.

C, Reverse Transcription PCR (RT-PCR) on total RNA extracts from ovaries. Electrophoretic separation of the RT-PCR reaction made on total RNA of flies *CG7009* homozygous or *CG7009* heterozygous for the mutant allele *CG7009*^{e02001} or no RT control (no reverse transcriptase in the RT reaction). The used primers for the PCR reaction are CG7009-middle Rev and CG7009-FW (expected product size 500nt) and tubulin primers (expected product size 150nt).

D, Reverse Transcription qPCR (RT-qPCR) on total RNA extracts from adult females of the indicated genotypes. *CG7009*^{e02001} heterozygous (Control), *CG7009*^{e02001} homozygous mutant (Mut CG7009) and rescue CG7009 (BAC).

E, Genotyping by PCR on genomic DNA of heterozygous *Def9487* and *Def3340* and *CG7009*^{e02001}. BAC (rescue) / CyO ; *CG7009*^{e02001} / *Def9487* lines, w1118 and *CG7009*^{e02001} homozygous lines. PCR on gDNA extracted from adult single flies with the indicated genotypes using primers CG7009-FW and CG7009-Rev. The band at 1148 bp corresponds to the WT *CG7009* locus, the band at 7119 bp corresponds to the mutant allele *CG7009*^{e02001}, containing the inserted *PiggyBac* transposon (Supplementary Figure S2A, B). BAC: Bacterial Artificial Chromosome containing the wild type *CG7009* genomic region; CyO; TM3,Sb; TM6,Tb,Sb: balancer chromosomes; Kbp: Kilo base pairs; gDNA genomic DNA.

F, Validation by sanger sequencing of the CRISPR/Cas9 mutants *CG5220*$^{K>A}$ and double mutant *CG5220*$^{K>A}$*, CG7009*^{e02001} recombination. Briefly, *CG5220* PCR fragments were amplified by PCR from flies gDNA bearing the *CG7009*^{e02001} allele (giving the [w+] phenotype) and the mutations *CG5220*$^{K>A}$. The corresponding simple mutant *CG5220*$^{K>A}$ (heterozygous for *CG5220*$^{K>A}$) was used as positive controls and flies characterized with no *CG5220* mutation were used as negative controls (*CG7009*^{e02001}). All sequencing experiments were performed on heteroallelic combinations over balanced chromosomes, explaining the double peaks at the edited region. The results were obtained using 4Peaks. They correspond to a PCR products obtained using VIE0197/VIE0198 primers and sequenced with the primer VIE0198. The targeted nucleotides are indicated under the red lines.

Figure S3.

A, RT-qPCR on ovaries heterozygous (control) or homozygous (Mut CG7009) for *CG7009*^{e02001} expressing the *Gypsy::LacZ* sensor (tj>Gal4/ +; Gypsy::LacZ) using *gyspy*- or *LacZ*- specific primers as described in Figure 2F. Error bars represent the standard deviation (s.d.) between three independent biological replicates. **p*-value < 0.05; ***p*-value < 0.01; ***p-value < 0.001 in a Student's t-test.

B, CG7009 and CG5220 are involved in *burdock* germinal TE-repression in *Drosophila* ovaries. *burdock::LacZ* sensor is silenced in germinal cells using nos>Gal4-mediated expression of an UAS-RNAi line (KD) targeting the *white* gene (KD control, nos>Gal4/ +; burdock::LacZ/UAS-*white*-RNAi). *Burdock* silencing is disrupted using *piwi* KD (positive control: blue coloration = β-Gal staining) and after KD of *CG7009* and *CG5220* expression.

C, RT-qPCR on ovaries from *w1118* (Control), homozygous for *CG7009*^{e02001} (Mut CG7009) or knockdown (Mut Moon), using *Roo, Invader1* or *R2* specific primers. Error bars represent the standard deviation (s.d.) between three to four independent biological replicates. **p*-value < 0.05; ***p*-value < 0.01; ***p-value < 0.001 in a Student's t-test.

Figure S4.

A, Sequence of *Drosophila melanogaster* tRNA$^{Phe(GAA)}$ with m/z values of fragments containing 2'-O-methyl-guanylate (#) and/or 2'-O-methyl-cytidylate (B) in daltons. The expected fragments resulting from RNase A (top) and RNase T1 (bottom) digestion of tRNA$^{Phe(GAA)}$ with the indicated Nm modification are zoomed in.

B, Schematic representation of 2D-structure of tRNA$^{Phe(GAA)}$ of *Drosophila*. Cm_{32} (B) and Gm_{34} (#) are indicated in yellow. **Bottom right**: Names of the different modifications present on *Drosophila* tRNA$^{Phe(GAA)}$ based on the Modomics and tRNAdb nomenclature**.**

Figure S5.

A, Related to Figures 5A and 5B. MALDI TOF-MS spectrum of fragments resulting from RNase A digestion of tRNA$^{Phe(GAA)}$ originating from indicated genotypes.

B, Related to Figures 5A and 5B. **Left panel,** MALDI TOF-MS spectrum of fragments resulting from RNase A digestion of tRNA$^{Phe(GAA)}$ originating from indicated genotypes (homozygous adult *CG7009*^{e02001} mutants rescued with one CG7009 WT copy (BAC)). **Right panel**: MALDI TOF-MS spectrum of fragments resulting from RNase T1 digestion of tRNA$^{Phe(GAA)}$ originating

from the indicated genotypes (homozygous adult *CG7009*^{e02001} mutants rescued with one CG7009 WT copy (BAC)). Relevant peaks are identified by their m/z values.

Figure S6.

A, Related to Figure 5C. RiboMethSeq analysis of tRNA$^{Phe(GAA)}$ modification at positions Cm_{32} and Gm_{34}. Alkaline fragmentation-based RiboMethSeq was performed on total RNAs extracted from whole flies homozygous mutant for *CG5220*$^{K>A}$, homozygous for *CG7009*^{e02001}/ *Def3340* and homozygous *CG5220*$^{K>A}$, *CG7009*^{e02001} double mutant as indicated. Normalized cleavage efficiencies, calculated from combined 5'-end and 3'-end coverages, are shown for the ± 5 neighboring nucleotides. The positions of interest (Cm_{32} and Gm_{34}) in tRNA$^{Phe(GAA)}$ are indicated by red arrows. Protection against cleavage is indicated as (+): protected, and as (-): not protected. Normalized cleavage efficiencies at Cm_{32} in *CG7009* mutant flies is moderate, indicating incomplete ribose methylation. A different visualisation for tRNA$^{Phe(GAA)}$ at position C32 and C34 is depicted in Figure S6B.

B, Related to Figure 5C and Supplementary Figure S6A. RiboMethSeq was performed as described in Supplementary Figure S6A for tRNA$^{Phe(GAA)}$ on 6 indicated genotypes. For a better visualization, raw read counts are presented in an non-normalized fashion (raw reads). The positions of interest (Cm_{32} and Gm_{34}) in tRNA$^{Phe(GAA)}$ are indicated by red arrows. Protection against cleavage is indicated as (+): protected and as (-): not protected. *CG7009*^{e02001}/+ mutants (Control), homozygous *CG5220*$^{K>A}$ mutant (*CG5220 K>A*), two independent genetic background mutants for *CG7009*: homozygous *CG7009*^{e02001} (*CG7009*^{e02001}) or trans-heterozygous *CG7009*^{e02001}/*Def3340* mutant (*CG7009*^{e02001}/*Def3340)*, double homozygous *CG7009*^{e02001},*CG5220*$^{K>A}$ mutant (*CG7009*^{e02001},*CG5220* K>A) and rescue BAC; *CG7009*^{e02001}/*Def3340* (Rescue CG7009 (BAC)).

C, RiboMethSeq was performed as described in Figure 5C and Supplementary Figure S6A for tRNA$^{Leu(CAA)}$ on 6 indicated genotypes. Normalized cleavage efficiencies, calculated from combined 5'-end and 3'-end coverages, are shown for the ± 5 neighboring nucleotides. The positions of interest (Cm_{32} and Gm_{34}) in tRNA$^{Leu(CAA)}$ are indicated by red arrows. Protection against cleavage is indicated as (+): protected and as (-): not protected. *CG7009*^{e02001}/+ mutants (Control), homozygous *CG5220*$^{K>A}$ mutant (*CG5220 K>A*), two independent genetic background mutants for *CG7009*: homozygous *CG7009*^{e02001} (*CG7009*^{e02001}) or trans-heterozygous *CG7009*^{e02001}/*Def3340* mutant (*CG7009*^{e02001}/*Def3340)*, double homozygous

CG7009^{e02001},*CG5220*$^{K>A}$ mutant (*CG7009*^{e02001},*CG5220* K>A) and rescue BAC; *CG7009*^{e02001}/*Def3340* (Rescue CG7009 (BAC)).

Figure S7.

Heatmap of normalized MethScores (ScoreC) for tRNA positions Cm/Um_{32} and Cm/Gm_{34} in different mutant backgrounds. Pink color corresponds to a high methylation compared to the average MethScores, blue or white color to lower methylation. Simplified genotypes are indicated in the middle (detailed genotypes in Figures S6B and S6C legends above), affected tRNA isoacceptors are given on the right. Scale of the color key (-1 to +1) is indicated at the top.
